# Radiomic score for lung nodules as a prognostic biomarker in locally advanced rectal cancer patients: A bi‐institutional study

**DOI:** 10.1002/cam4.7240

**Published:** 2024-06-24

**Authors:** Zhiyuan Zhang, Jiazhou Wang, Di Dai, Fan Xia, Yiqun Sun, Guichao Li, Juefeng Wan, Lijun Shen, Hui Zhang, Yan Wang, Jie Zhong, Jun Bao, Zhen Zhang

**Affiliations:** ^1^ Department of Radiation Oncology Fudan University Shanghai Cancer Center Shanghai China; ^2^ Department of Oncology, Shanghai Medical College Fudan University Shanghai China; ^3^ Shanghai Clinical Research Center for Radiation Oncology Shanghai China; ^4^ Shanghai Key Laboratory of Radiation Oncology Shanghai China; ^5^ Department of Radiology Nanjing Medical University Affiliated Cancer Hospital, Jiangsu Cancer Hospital, Jiangsu Institute of Cancer Research Nanjing China; ^6^ Shanghai Institute of Medical Imaging Fudan University Shanghai China; ^7^ Department of Radiology Fudan University Shanghai Cancer Center Shanghai China; ^8^ Department of Oncology Nanjing Medical University Affiliated Cancer Hospital, Jiangsu Cancer Hospital, Jiangsu Institute of Cancer Research Nanjing China

**Keywords:** locally advanced rectal cancer, lung nodules, prognostic prediction, radiomics

## Abstract

**Background:**

Undetermined lung nodules are common in locally advanced rectal cancer (LARC) and lack precise risk stratification. This study aimed to develop a radiomic‐based score (Rad‐score) to distinguish metastasis and predict overall survival (OS) in patients with LARC and lung nodules.

**Methods:**

Retrospective data from two institutions (July 10, 2006—September 24, 2015) was used to develop and validate the Rad‐score for distinguishing lung nodule malignancy. The prognostic value of the Rad‐score was investigated in LARC cohorts, leading to the construction and validation of a clinical and radiomic score (Cli‐Rad‐score) that incorporates both clinical and radiomic information for the purpose of improving personalized clinical prognosis prediction. Descriptive statistics, survival analysis, and model comparison were performed to assess the results.

**Results:**

The Rad‐score demonstrated great performance in distinguishing malignancy, with C‐index values of 0.793 [95% CI: 0.729–0.856] in the training set and 0.730 [95% CI: 0.666–0.874] in the validation set. In independent LARC cohorts, Rad‐score validation achieved C‐index values of 0.794 [95% CI: 0.737–0.851] and 0.747 [95% CI: 0.615–0.879]. Regarding prognostic prediction, Rad‐score effectively stratified patients. Cli‐Rad‐score outperformed the clinicopathological information alone in risk stratification, as evidenced by significantly higher C‐index values (0.735 vs. 0.695 in the internal set and 0.618 vs. 0.595 in the external set).

**Conclusions:**

CT‐based radiomics could serve as a reliable and powerful tool for lung nodule malignancy distinction and prognostic prediction in LARC patients. Rad‐score predicts prognosis independently. Incorporation of Cli‐Rad‐score significantly enhances the persionalized clinical prognostic capacity in LARC patients with lung nodules.

## 1 INTRODUCTION

Tumor metastasis is a significant contributor to mortality in colorectal cancer (CRC). Among the extra‐abdominal sites, lung metastases are more commonly observed in rectal cancer compared to colon cancer.^1^, ^2^, ^3^ Accurately diagnosing lung nodules plays a crucial role in the management of locally advanced rectal cancer (LARC) as it aids in determining tumor stage and guiding personalized treatment decisions. In the era of precision and personalized medicine, various clinical approaches have been suggested to evaluate the malignancy of lung nodules and assess the risk of prognosis.^4^, ^5^, ^6^ However, confirming the nature of lung nodules poses challenges due to the invasive nature of biopsies or the need for prolonged follow‐up, which is often impractical during the initial diagnostic phase. Imaging features and PET‐CT scans can provide indications of potentially malignant nodules. For high‐risk patients, it may also be advisable to perform biopsies to accurately determine the pathological type of the nodules. Due to the challenges associated with confirming the nature of benign nodules, a long‐term follow‐up approach is often employed in clinical practice. This is inappropriate as clinically suspected benign nodules undergo surgical confirmation. While this may not be the ideal solution, it represents the closest approximation to the “gold standard” currently utilized in the field. Therefore, the identification of reliable indicators for recognizing malignant lung nodules and assessing prognostic risk is of utmost importance. Radiomics is a sophisticated quantitative tool that transforms digital medical images into high‐dimensional data, allowing researchers to extract quantitative features.^7^ Previous studies on radiomics have demonstrated its significant potential in identifying malignancies and classifying prognostic outcomes in CRC patients.^8^, ^9^ However, the value of radiomics in prognostic prediction for LARC patients is an area that requires further investigation and exploration.

There are numerous prognostic prediction models for locally progressive rectal cancer, and in addition to conventional clinical factors, it is important to explore imaging features. Image‐based predictive tools come from several main sources: advances in model building with multi‐omics, functional magnetic resonance image sequence, and incorporation of additional predictors such as PETCT.^10^, ^11^, ^12^ However, all these predictions are aimed at primary foci for affective feature extraction, and there is still a lack of appropriate assessment methods for patients at high risk of potential lung metastases accompanied by pulmonary nodules.

Our study aimed to evaluate the significance of CT‐based radiomic features in identifying lung metastases and predicting prognosis in patients with LARC.

## 2 MATERIALS AND METHODS

### 2.1 Study design and data source

In this retrospective study, we recruited CRC and LARC patients from two hospitals: Fudan University Shanghai Cancer Center (FUSCC) and JiangSu Province Cancer Center (JSCC). The development of the predictive model for the lung nodule radiomic score (Rad‐score) was based on a dataset of 235 CRC patients from FUSCC. Benign lung nodules were confirmed by stable morphological state on follow‐up CT scans over a 2‐year period or through pathological biopsy. Lung metastases were verified through surgical resection or biopsy specimens. The CRC patient cohort was randomly divided into two datasets, Rad‐train and Rad‐valid, with a 70/30 ratio, respectively, for training and validation using a random algorithm. After constructing the model, we applied the Rad‐score to validate the nodules nature assessment of malignancy in patients with LARC. Internal validation was performed using LARC patients from FUSCC (FUSCC cohort). Information regarding lung nodules was collected from follow‐up data and pathological diagnoses. Following internal validation, we recruited LARC patients from JSCC for external validation (JSCC cohort). The data from these different cohorts were meticulously separated to ensure the rigorous validation of the model.

**FIGURE 1:**
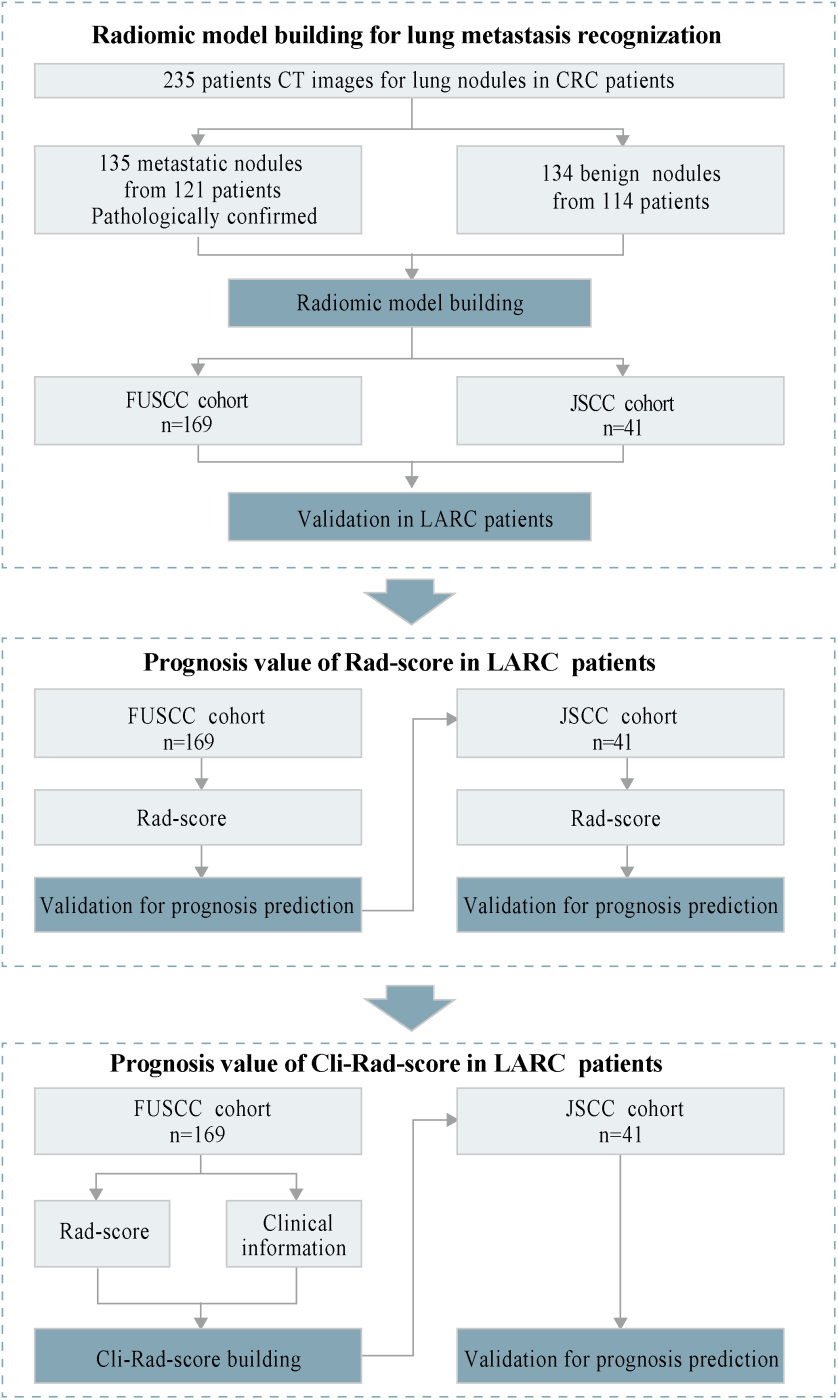
Flowchart for radiomic study. The lung images from CRC patients in Fudan University Shanghai Cancer Center were divided into two cohorts at a ratio of 7:3 to generate a radiomic model by LASSO regression and validate it. To test the stability of the model in LARC patients, the malignant prediction from the radiomic model was conducted in two separate cohorts from FUSCC (FUSCC cohort) and JiangSu province Cancer Center (JSCC cohort). To explore the prognostic value of the radiomic model in LARC patients, a COX regression model was built based on LARC patients in FUSCC, and then a nomogram based on the clinical and radiomic information was built in FUSCC cohort and validated in JSCC cohort. CRC colon and rectal cancer, FUSCC, Fudan University Shanghai Cancer Center; JSCC, JiangSu province Cancer Center; LARC Locally Advanced Rectal Cancer; LASSO, Least Absolute Shrinkage and Selection Operator.

To predict prognosis, relevant clinical information was extracted from the electronic medical record (EMR) system. Independent clinical prognostic factors were identified through careful filtering. To further evaluate the enhanced prognostic prediction capacity of established Rad‐score, Cli‐Rad‐score was developed based on a nomogram with Rad‐score and clinical prognostic factord. The Cli‐Rad‐score was constructed using the FUSCC cohort and further validated in the JSCC cohort. Survival data obtained from medical records or follow‐ups conducted by healthcare professionals, were utilized for analysis. Please refer to Figure 1; Figure S1 for visual representation of the study flow diagram.

This study received approval from the Institutional Review Boards of both FUSCC and JSCC. It was conducted in accordance with the ethical principles outlined in the 1964 Helsinki Declaration and its subsequent amendments.

### 2.2 Patients' enrollment and exclusion criteria

To establish the Rad‐score, a group of CRC patients was selected based on specific criteria. These criteria included: (1) Histologically‐confirmed colorectal cancer without any other concurrent malignant tumors, (2) histologically‐confirmed lung metastases in the period of follow‐up, (3) follow‐up of non‐progressing lesions for at least 2 years or biopsy confirmation for benign nodules, (4) presence of at least one pulmonary nodule with a diameter less than 20 mm, (5) inclusion of only solid nodules. It is important to note that no contrast injection was administered to the patients in this cohort.

For the prognostic analysis, the inclusion criteria for rectal cancer patients were as follows: (1) histologically‐confirmed locally advanced rectal cancer without any other concurrent malignant tumors, (2) histologically‐proven lung metastases in the period of follow‐up or evidence of increasing volume during the follow‐up period, (3) detection of lung metastases within 6 months from the time of diagnosis, (4) follow‐up of non‐progressing lesions for at least 1 year or biopsy confirmation for benign nodules, (5) presence of at least one pulmonary nodule with a diameter less than 20 mm, (6) availability of complete CT datasets and clinical characteristics, (7) inclusion of only solid nodules. It is important to note that no contrast injection was administered to the patients in this analysis. The overall survival (OS) time was defined from the date of diagnosis to the date of death event.

### 2.3 CT image segmentation and radiomic model development

All patients included in the study underwent lung CT examinations prior to neoadjuvant chemoradiotherapy or surgery. In cases where patients had multiple nodules, we randomly selected up to three qualified nodules for inclusion. The selected nodule lesion was then reconstructed using standard parameters, including a slice thickness of 1.0 mm, slice increment of 1 mm, pitch of 1.078, field of view of 15 cm, and a matrix size of 512 × 512. These parameters were used to ensure consistent and accurate image reconstruction for further analysis.

CT scan images were retrieved from the hospital's picture archiving and communication system (PACS). In order to analyze the images, the boundaries of the regions of interest (ROIs) were defined by the outer contour of the solid pulmonary nodules. These ROIs were then composed into a 3D volume, with each slide representing a slice of the ROI. A doctor with at least 5 years of experience in interpreting medical images delineates the boundaries, which are then reviewed by another doctor with at least 10 years of experience using MIM software (v6.6.3; MIM Software Inc.). Figure S2 provides representative examples of this process.

A total of 254 radiomic features were extracted from the CT scan images using a custom MATLAB‐based tool (MathWorks, Natick, MA, USA). Our previous publications^13^ provide a detailed description of the extraction algorithms utilized in this study, which categorized the extracted features into four groups: (1) histogram‐based features, (2) gray level co‐occurrence matrix (GLCM) features,^14^ (3) gray‐level run‐length matrix (GLRLM) features,^15^ and (4) wavelet‐based features. To identify the most informative features, the least absolute shrinkage and selection operator (LASSO) logistic model was employed in the training cohort. Subsequently, a logistic model was utilized to establish the Rad‐score based on the selected features.

### 2.4 Evaluation of prognostic prediction score

Furthermore, we assessed the prognostic predictive ability of the Rad‐score in LARC patients with lung nodules. Univariate Cox analysis was conducted to evaluate the association between the Rad‐score and patient outcomes. To determine the optimal cutoff value for the Rad‐score, we utilized the algorithm provided by the “survminer” package in R.

### 2.5 Prognostic model establishment and validation

The EMR system was utilized to obtain clinical and pathological information regarding patients. This data included age, gender, primary tumor location, type of surgery, distribution and number of pulmonary nodules, levels of carcinoembryonic antigen (CEA), clinical stage (cTNM), pathological stage after surgery (ypTNM), pathological type, response to neoadjuvant chemotherapy, degree of tumor differentiation, presence of vascular invasion, perineural invasion (PNI), circumferential resection margin (CRM), and patient survival outcomes.

Univariate and multivariate Cox regression analyses were performed to identify potential predictors of survival among the collected clinicopathological variables. Variables that displayed statistical significance (*p* < 0.05) in the multivariate Cox regression analysis were integrated into the development of the prognostic Cli‐Rad‐score, in conjunction with the Rad‐score. The discriminatory ability of the prognostic Cli‐Rad‐score and the Rad‐score biomarker was assessed using Harrell's concordance index (C‐index) and Kaplan–Meier (KM) survival analysis.

### 2.6 Statistical analysis

All statistical analyses were performed using R software (version 4.0.1; https://www.r‐project.org/). Two‐sided tests were conducted, and a significance level of *p* < 0.05 was considered statistically significant. The following R packages were utilized: “glmnet”, “timeROC”, “rms”, ‘survival’, “Hmisc”, and “maxstat.” LASSO regression was employed for feature selection, model building, and the calculation of receiver operating characteristic (ROC) curves and C‐indexes to evaluate model performance. Survival analysis was conducted using a Cox proportional hazards model. KM survival analysis, log‐rank test and C‐index were employed to assess OS prediction. ANOVA test was used to compare the time‐related predictive ability of the prognostic models. The optimal cutoff point was determined using the “maxstat” package. Calibration curves for the models can be found in the supplementary materials.

## 3 RESULTS

### 3.1 Patient characteristics

A radiomics model was developed using 235 CRC patients, divided into a training set (Rad‐train) and a validation set (Rad‐valid). In the Rad‐train set, there were 89 benign lung nodules and 84 metastatic nodules. The diagnosis of benign nodules was primarily established through follow‐up data, with a median follow‐up duration of 60.2 months implemented to ensure the reliability of the diagnosis. Detailed patient characteristics can be found in Table 1, while specific information for the Rad‐train and Rad‐valid sets is provided in Tables S2 and S3.

**TABLE 1:** Demographic and clinical characteristics of patients enrolled for LARC cohorts.

	Patients enrolled in LARC in FUSCC			*p*	Patients enrolled in LARC in JSCC			*p*
		Nodules distribution				Nodules distribution		
	Overall (*n* = 169)	Benign (*n* = 96)	Metastasis (*n* = 73)		Overall (*n* = 41)	Benign (*n* = 26)	Metastasis (*n* = 15)	
Gender, *n* (%)								
Female	62 (36.7)	34 (35.4)	28 (38.4)	0.817	29 (70.7)	18 (69.2)	11 (73.3)	1.000
Male	107 (63.3)	62 (64.6)	45 (61.6)		12 (29.3)	8 (30.8)	4 (26.7)	
Age, Median (IQR)	57 [48.00, 62.00]	55.5 [47.00, 61.00]	58 [50.00, 62.00]	0.124	56.56 (11.09)	57.96 (11.21)	54.13 (10.82)	0.293
Location, *n* (%)								
Rectal	169 (100.0)	96 (100.0)	73 (100.0)		41 (100.0)	26 (100.0)	15 (100.0)	
Colon	0 (0.0)	0 (0.0)	0 (0.0)		0 (0.0)	0 (0.0)	0 (0.0)	
cT, *n* (%)								
cT2	4 (2.4)	4 (4.2)	0 (0.0)	0.218	1 (2.4)	0 (0.0)	1 (6.7)	0.180
cT3	134 (79.3)	76 (79.2)	58 (79.5)		16 (39.0)	12 (46.2)	4 (26.7)	
cT4	31 (18.3)	16 (16.7)	15 (20.5)		24 (58.5)	14 (53.8)	10 (66.7)	
cN, *n* (%)								
cN0	9 (5.3)	5 (5.2)	4 (5.5)	0.459	9 (22.0)	4 (15.4)	5 (33.3)	0.200
cN1	60 (35.5)	38 (39.6)	22 (30.1)		14 (34.1)	8 (30.8)	6 (40.0)	
cN2	100 (59.2)	53 (55.2)	47 (64.4)		18 (43.9)	14 (53.8)	4 (26.7)	
cstage, *n* (%)								
cI	2 (1.2)	2 (2.1)	0 (0.0)	0.529	0 (0.0)	0 (0.0)	0 (0.0)	0.017
cII	7 (4.1)	3 (3.1)	4 (5.5)		7 (17.1)	4 (15.4)	3 (20.0)	
cIII	160 (94.7)	91 (94.8)	69 (94.5)		30 (73.2)	22 (84.6)	8 (53.3)	
cIV	0 (0.0)	0 (0.0)	0 (0.0)		4 (9.8)	0 (0.0)	4 (26.7)	
CEA, *n* (%)								
Normal	91 (53.8)	61 (63.5)	30 (41.1)	0.001	36 (87.8)	22 (84.6)	14 (93.3)	0.744
Abnormal	75 (44.4)	32 (33.3)	43 (58.9)		5 (12.2)	4 (15.4)	1 (6.7)	
Unknown	3 (1.8)	3 (3.1)	0 (0.0)		0 (0.0)	0 (0.0)	0 (0.0)	
Distance to anus, *n* (%)								
>5 cm	74 (43.8)	50 (52.1)	24 (32.9)	0.019	30 (73.2)	18 (69.2)	12 (80.0)	0.701
<5 cm	95 (56.2)	46 (47.9)	49 (67.1)		11 (26.8)	8 (30.8)	3 (20.0)	
Neoadjuvant treatment, *n* (%)								
Yes	169 (100.0)	96 (100.0)	73 (100.0)		26 (63.4)	16 (61.5)	10 (66.7)	1.000
No	0 (0.0)	0 (0.0)	0 (0.0)		15 (36.6)	10 (38.5)	5 (33.3)	
Surgery, *n* (%)								
No	0 (0.0)	0 (0.0)	0 (0.0)	0.149	3 (7.3)	1 (3.8)	2 (13.3)	0.164
Dixon	91 (53.8)	58 (60.4)	33 (45.2)		23 (56.1)	17 (65.4)	6 (40.0)	
Miles	72 (42.6)	35 (36.5)	37 (50.7)		13 (31.7)	6 (23.1)	7 (46.7)	
Colon cancer resection	0 (0.0)	0 (0.0)	0 (0.0)		0 (0.0)	0 (0.0)	0 (0.0)	
Transanal resection	0 (0.0)	0 (0.0)	0 (0.0)		0 (0.0)	0 (0.0)	0 (0.0)	
Hartmann	6 (3.6)	3 (3.1)	3 (4.1)		2 (4.9)	2 (7.7)	0 (0.0)	
pCR, *n* (%)								
Non‐pCR	142 (84.0)	73 (76.0)	69 (94.5)	<0.001	5 (12.2)	4 (15.4)	1 (6.7)	0.636
pCR	24 (14.2)	22 (22.9)	2 (2.7)		36 (87.8)	22 (84.6)	14 (93.3)	
Unknown	3 (1.8)	1 (1.0)	2 (2.7)		0 (0.0)	0 (0.0)	0 (0.0)	
Pathology, *n* (%)								
Adenocarcinoma	159 (94.1)	87 (90.6)	72 (98.6)	0.097	35 (85.4)	22 (84.6)	13 (86.7)	0.895
Mucinous adenocarcinoma	4 (2.4)	4 (4.2)	0 (0.0)		2 (4.9)	1 (3.8)	1 (6.7)	
Signet ring cell carcinoma	0 (0.0)	0 (0.0)	0 (0.0)		2 (4.9)	2 (7.7)	0 (0.0)	
Squamous	0 (0.0)	0 (0.0)	0 (0.0)		2 (4.9)	1 (3.8)	1 (6.7)	
Unknown	6 (3.6)	5 (5.2)	1 (1.4)		0 (0.0)	0 (0.0)	0 (0.0)	
Differentiation, *n* (%)								
Poorly	17 (10.1)	5 (5.2)	12 (16.4)	<0.001	14 (34.1)	8 (30.8)	6 (40.0)	0.637
Moderately	82 (48.5)	39 (40.6)	43 (58.9)		18 (43.9)	11 (42.3)	7 (46.7)	
Highly	4 (2.4)	3 (3.1)	1 (1.4)		2 (4.9)	1 (3.8)	1 (6.7)	
Unknown	66 (39.1)	49 (51.0)	17 (23.3)		7 (17.1)	6 (23.1)	1 (6.7)	
CRM, *n* (%)								
Negative	165 (97.6)	96 (100)	69 (94.5)	0.033	26 (63.4)	14 (53.8)	12 (80.0)	0.181
Positive	4 (2.4)	0 (0.0)	4 (5.5)		0 (0.0)	0 (0.0)	0 (0.0)	
Unknown	0 (0.0)	0 (0.0)	0 (0.0)		15 (36.6)	12 (46.2)	3 (20.0)	
Vascular invasion, *n* (%)								
Negative	155 (91.7)	91 (94.8)	64 (87.7)	0.167	22 (53.7)	12 (46.2)	10 (66.7)	0.344
Positive	14 (8.3)	5 (5.2)	9 (12.3)		5 (12.2)	3 (11.5)	2 (13.3)	
Unknown	0 (0.0)	0 (0.0)	0 (0.0)		14 (34.1)	11 (42.3)	3 (20.0)	
Perineural invasion, *n* (%)								
Negative	144 (85.2)	90 (93.8)	54 (74.0)	0.001	24 (58.5)	14 (53.8)	10 (66.7)	0.196
Positive	25 (14.8)	6 (6.2)	19 (26.0)		3 (7.3)	1 (3.8)	2 (13.3)	
Unknown	0 (0.0)	0 (0.0)	0 (0.0)		14 (34.1)	11 (42.3)	3 (20.0)	
ypT, *n* (%)								
ypT0	29 (17.2)	26 (27.1)	3 (4.1)	<0.001	8 (19.5)	7 (26.9)	1 (6.7)	0.489
ypT1	6 (3.6)	5 (5.2)	1 (1.4)		2 (4.9)	1 (3.8)	1 (6.7)	
ypT2	45 (26.6)	25 (26.0)	20 (27.4)		11 (26.8)	6 (23.1)	5 (33.3)	
ypT3	86 (50.9)	39 (40.6)	47 (64.4)		15 (36.6)	10 (38.5)	5 (33.3)	
ypT4	3 (1.8)	1 (1.0)	2 (2.7)		2 (4.9)	1 (3.8)	1 (6.7)	
Unknown	0 (0.0)	0 (0.0)	0 (0.0)		3 (7.3)	1 (3.8)	2 (13.3)	
ypN, *n* (%)								
ypN0	103 (60.9)	69 (71.9)	34 (46.6)	0.003	32 (78.0)	20 (76.9)	12 (80.0)	0.708
ypN1	52 (30.8)	20 (20.8)	32 (43.8)		7 (17.1)	4 (15.4)	3 (20.0)	
ypN2	14 (8.3)	7 (7.3)	7 (9.6)		2 (4.9)	2 (7.7)	0 (0.0)	
Unknown	0 (0.0)	0 (0.0)	0 (0.0)		0 (0.0)	0 (0.0)	0 (0.0)	
yp‐stage, *n* (%)								
yp0	24 (14.2)	22 (22.9)	2 (2.7)	<0.001	11 (26.8)	9 (34.6)	2 (13.3)	0.516
ypI	38 (22.5)	27 (28.1)	11 (15.1)		11 (26.8)	6 (23.1)	5 (33.3)	
ypII	39 (23.1)	19 (19.8)	20 (27.4)		7 (17.1)	4 (15.4)	3 (20.0)	
ypIII	68 (40.2)	28 (29.2)	40 (54.8)		9 (22.0)	6 (23.1)	3 (20.0)	
Unknown	0 (0.0)	0 (0.0)	0 (0.0)		3 (7.3)	1 (3.8)	2 (13.3)	
Pathologic confirmation, *n* (%)								
No	139 (82.2)	94 (97.9)	45 (61.6)	<0.001	40 (97.6)	26 (100.0)	14 (93.3)	0.366
Yes	30 (17.8)	2 (2.1)	28 (38.4)		1 (2.4)	0 (0.0)	1 (6.7)	
Number of lung nodules, *n* (%)								
Single	87 (51.5)	63 (65.6)	24 (32.9)	<0.001	15 (36.6)	12 (46.2)	3 (20.0)	0.181
Multi	82 (48.5)	33 (34.4)	49 (67.1)		26 (63.4)	14 (53.8)	12 (80.0)	
Lateral, *n* (%)								
Unilateral	103 (60.9)	70 (72.9)	33 (45.2)	<0.001	22 (53.7)	17 (65.4)	5 (33.3)	0.097
Bilateral	66 (39.1)	26 (27.1)	40 (54.8)		19 (46.3)	9 (34.6)	10 (66.4)	

*Note*: The *p*‐value for age was 0.679 from the Shapiro–Wilk normality test, which tests the normal distribution of age.

Abbreviations: CEA, carcinoma embryonic antigen; cN, clinical nodal; CRM, circumferential resection margin; cT, clinical tumor; FUSCC, Fudan University Cancer Center; JSCC, JiangSu province Cancer Center; LARC, Locally Advanced Rectal Cancer; pCR, pathological complete regression; ypN, pathological lymph node stage after neoadjuvant therapy; ypT, pathological tumor T stage after neoadjuvant therapy.

To validate the model's performance in predicting malignancy in LARC patients, a total of 238 nodules from 169 patients in the FUSCC cohort and 63 nodules from 41 patients in the JSCC cohort were included. Among these, 96 nodules in the FUSCC cohort and 26 nodules in the JSCC cohort were malignant. Due to the size of some nodules, it may not always be possible to obtain pathological confirmation, particularly in cases where the patient is diagnosed with locally advanced rectal cancer. However, in the FUSCC cohort, pathological confirmation was available for 17.8% of patients, and in the JSCC cohort, it was available for 2.4% of patients. In most cases, nodules were confirmed through follow‐up. The median follow‐up times for LARC patients were 58.4 months in the FUSCC cohort and 31.4 months in the JSCC cohort. Detailed information can be found in Table 1; Table S1.

### 3.2 Rad‐score construction and validation

The Rad‐score matrix was derived from the gross tumor volume (GTV) calculated from CT images. Through the utilization of least absolute shrinkage and selection operator (LASSO) regression (Figure S3), the Rad‐score was constructed using seven selected radiomic features: “scaled_kurtosis,” “LL_GLCM_Maximal_Correlation_Coefficient,” “HL_GLCM_Sum_variance,” “LL_GLRMS_LRHGE,” “HH_GLRMS_LGRE,” “LH_absolute_median,” and “HL_absolute_median.” These features, listed in Table S4, capture the heterogeneity characteristics of malignant nodules, which represent more aggressive tumor variants with chaotic and disordered growth patterns.

**FIGURE 2:**
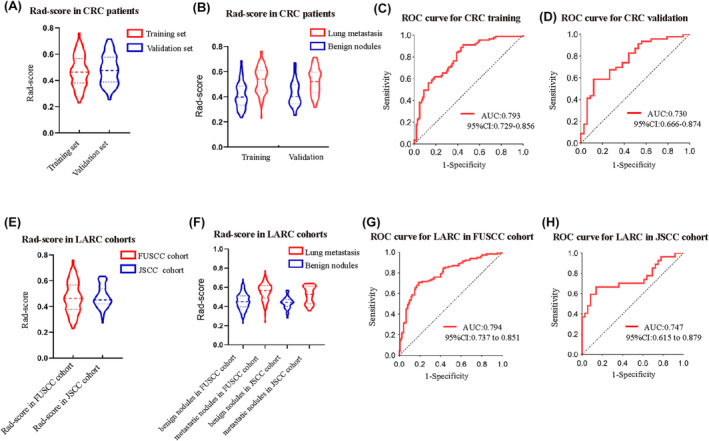
Training and validation for malignant prediction. The radiomic model was established in cohorts of CRC patients in FUSCC, whose lung metastasis was confirmed by pathological examination. (A, B) represents the Rad‐score from CRC patients. For further validation in LARC patients, patients from FUSCC and JSCC were enrolled, and their lung nodules were extracted for radiomic prediction. (C, D) represent the Rad‐score and concordance in the training set and validation set of CRC patients from FUSCC, with the cut‐off value of 0.405 and 0.518, respectively. (E, F) represents the Rad‐score of LARC patients' lung nodules from FUSCC and JSCC. (G, H) represent the Rad‐score and concordance from LARC patients in FUSCC and JSCC separately, with the cut‐off value of 0.518 and 0.509, respectively. CRC colon and rectal cancer, FUSCC, Fudan University Shanghai Cancer Center; JSCC, JiangSu province Cancer Center; LARC, Locally Advanced Rectal Cancer.

Following the evaluation of the model, nodules were categorized into high‐risk and low‐risk groups based on an optimal cutoff value. Figure S4; Table S5 provide details about the distribution characteristics and cutoff values. The training and validation sets were constructed using data from CRC patients. In evaluating the model's performance in predicting lung metastasis, the areas under the curve (AUC) were 0.793 (95% CI: 0.729–0.856) for the Rad‐train set and 0.730 (95% CI: 0.666–0.874) for the Rad‐valid set, as illustrated in Figure 2.

### 3.3 Validation of the Rad‐score for lung metastasis distinction in LARC patients

In order to confirm the effectiveness of the Rad‐score in LARC patients, we conducted evaluations in two distinct cohorts: the LARC FUSCC cohort and the JSCC cohort. In the FUSCC cohort, the Rad‐score exhibited an AUC of 0.794 (95% CI: 0.737–0.851) for predicting lung metastasis, indicating a robust ability to differentiate between malignant and benign lung nodules. Similarly, in the JSCC cohort, the AUC for the Rad‐score prediction of lung metastasis was 0.747 (95% CI: 0.615–0.879). These results highlight the consistent and reliable performance of the Rad‐score in identifying malignant lung nodules in LARC patients. The features of the Rad‐score distribution are depicted in Figure 2, as well as in Table S5; Figure S4.

### 3.4 Prognostic value of the Rad‐score

**FIGURE 3:**
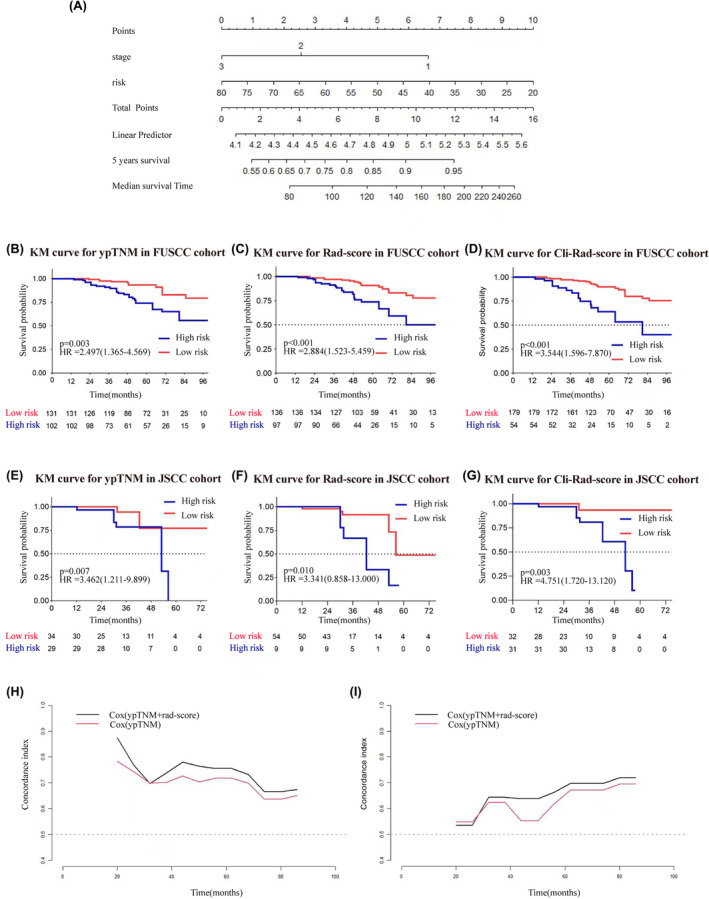
Survival analysis of ypTNM stage and Cli‐Rad‐score. (A) represents the nomogram for prognostic prediction, (B) represents the prognostic stratification from ypTNM stage in FUSCC, (C) represents the prognostic stratification from the Rad‐score in FUSCC, (D) represents the prognostic stratification from the Cli‐Rad‐score in FUSCC, (E) represents the prognostic stratification from ypTNM stage in JSCC, (F) represents the prognostic stratification from the Rad‐score in JSCC, (G) represents the prognostic stratification from the Cli‐Rad‐score in JSCC, (H) represents the incremental C‐index of the Cli‐Rad‐score compared to ypTNM stage in FUSCC, (I) represents the incremental C‐index of the Cli‐Rad‐score compared to ypTNM stage in JSCC.

The Rad‐score demonstrated a strong predictive ability for identifying LARC patients with poorer prognosis. KM analysis revealed a significant association between the Rad‐score and OS risk in both the FUSCC cohort (HR = 2.884, 95% CI: 1.523–5.459, *p* < 0.001) and JSCC cohort (HR = 3.341, 95% CI: 0.858–13.000, *p* = 0.010). Notably, patients with a low Rad‐score had longer OS compared to those with a high Rad‐score. More detailed information were presented in Figure 3C,F. Table 2 contains the C‐indices for the models, and additional details can be found in Table S8.

**TABLE 2:** C‐index for models.

	FUSCC cohort		JSCC cohort	
Model	C‐index	95% confidence interval	C‐index	95% confidence interval
Rad‐score for metastasis recognization in LARC patients	0.794	0.737–0.851	0.747	0.615–0.879
Prognosis prediction from ypTNM stage	0.695	0.638–0.752	0.595	0.472–0.718
Prognosis prediction from Rad‐score	0.633	0.538–0.727	0.546	0.412–0.680
Prognosis prediction from Cli‐Rad‐score	0.735	0.668–0.802	0.618	0.500–0.736

### 3.5 Construction of a comprehensive clinical and radiomic model

Following the completion of both univariate and multivariate Cox regression analyses, it was determined that only ypTNM stage after neoadjuvant chemoradiation was independently associated with prognosis (*p* = 0.005). These results were listed in Tables S6, S7. A nomogram was developed for the Cli‐Rad‐score, incorporating ypTNM stage and Rad‐score, as depicted in Figure 3A. Due to the limited number and similar clinical prognosis of patients with ypTNM 0‐I, they were combined for further analysis.

A multivariate Cox analysis was performed to investigate whether ypTNM and Rad‐score are independent prognostic factors. The KM analysis demonstrated a remarkably significant disparity in OS between the high‐risk and low‐risk cohorts, as stratified by the ypTNM stage (hazard ratio [HR] = 2.497, 95% confidence interval [CI]: 1.365–4.569, *p* = 0.003) and Cli‐Rad‐score (HR = 3.544, 95% CI: 1.596–7.870, *p* < 0.001) in the FUSCC cohort. Additionally, in the JSCC cohort, the ypTNM stage (HR = 3.462, 95% CI: 1.211–9.899, *p* = 0.007) and Cli‐Rad‐score (HR = 4.751, 95% CI: 1.720–13.120, *p* = 0.003) also showed a significant difference in OS (Figure 3B–G; Table S8). Calibration curves for all prognostic models can be found in Figure S5 in the supplementary materials.

The results of this study reveal that incorporating the Rad‐score into the prognosis prediction for patients with pulmonary nodules leads to increased accuracy.

### 3.6 Incremental value of the radiomic signature

Subsequently, we conducted an analysis to assess the extent of the Rad‐score's enhanced predictive capacity by evaluating the Cli‐Rad‐score. Table 2 displays the C‐index values for ypTNM stage, Rad‐score, and Cli‐Rad‐score. The Cli‐Rad‐score demonstrated a higher C‐index than ypTNM stage alone or Rad‐score alone in both cohorts (FUSCC cohort: C‐index = 0.735, 95% CI: 0.668–0.802; JSCC cohort: C‐index = 0.618, 95% CI: 0.500–0.736; ypTNM stage alone: FUSCC cohort: C‐index = 0.695, 95% CI: 0.538–0.727; JSCC cohort: C‐index = 0.595, 95% CI: 0.472–0.718; Rad‐score alone: FUSCC cohort: C‐index = 0.633, 95% CI: 0.538–0.727; JSCC cohort: C‐index = 0.546, 95% CI: 0.412–0.680).

An evaluation of the Cli‐Rad‐score's performance at various time points was conducted by time‐related C‐indexs. The Cli‐Rad‐score consistently exhibited a higher C‐index for prognostic prediction at different time points (Figure 3H,I). Anova test results indicated that the Cli‐Rad‐score significantly outperformed ypTNM stage alone in prognostic prediction (FUSCC cohort: *p* = 0.015, JSCC cohort: *p* < 0.001). These findings indicated that the Cli‐Rad‐score performed more effectively in prognostic prediction than ypTNM stage alone.

## 4 DISCUSSION

This study introduces a CT‐based radiomics score for predicting lung nodule nature and assessing survival in LARC patients. The Rad‐score, comprised of seven radiomic features, achieved high accuracy in identifying malignant nodules (AUC = 0.794, 95%CI: 0.737–0.851). It also showed good prognostic prediction performance in both the internal and external cohorts. Compared to ypTNM stage alone, the Cli‐Rad‐score demonstrated improved prognostic capability in the internal (C‐index = 0.735, 95%CI: 0.668–0.802, *p* = 0.015) and external cohorts (C‐index = 0.618, 95%CI: 0.500–0.736, *p* < 0.001). Radiomics holds promise as a reliable tool for distinguishing lung nodule malignancy and predicting prognosis in LARC patients.

Despite advances in neoadjuvant radiochemotherapy, metastasis remains a significant challenge in LARC patients, particularly when it comes to lung metastasis.^2^, ^16^ The high incidence of lung metastasis in this population, coupled with the difficulty of distinguishing it from benign nodules, often results in delays in receiving appropriate treatment.^3^, ^17^, ^18^, ^19^ This presents a critical clinical challenge in accurately evaluating the nature of lung nodules in LARC patients.

The use of early local treatment such as surgery and radiotherapy, for lung metastasis in selective patients has been the focus of extensive research and attention.^3^, ^20^, ^21^, ^22^, ^23^, ^24^, ^25^, ^26^, ^27^, ^28^, ^29^ However, there is currently limited conclusive evidence regarding the identification of biomarkers for screening high‐risk patients with a poor prognosis.^26^, ^30^, ^31^ The application of radiomics in the assessment of pulmonary nodules can provide valuable clinical insights for the identification of high‐risk patients. CT‐based radiomics facilitates clinical decision‐making with rich information.^29^, ^30^ Ongoing research explores conventional and dynamic radiomic features to detect lung metastasis.^14^, ^15^, ^31^ However, more exploration is required to fully understand the value of radiomic scores in LARC patients.

Our study had several limitations. First, the classification of pulmonary nodules in patients with LARC was mainly based on follow‐up data rather than pathological verification. Second, there is a scarcity of detailed information on adjuvant therapy and subsequent chemotherapy because of information loss while receiving treatment at various centers. Third, despite the possibility of employing other modeling techniques for a more comprehensive analysis, LASSO performed satisfactorily in our study. Finally, the external cohort comprised a restricted number of patients, and the inclusion of multiple nodules in some individuals may have impacted the statistical analysis. Future research should endeavor to overcome these limitations by accumulating more data and validating our findings in larger cohorts. In conclusion, our study validated the Rad‐score as a noninvasive method for assessing lung nodules malignancy in LARC patients. Additionally, we developed and validated the Cli‐Rad‐score prognostic model by integrating the Rad‐score and ypTNM staging. This research provides the initial assessment of the Rad‐score in predicting malignant tumors and prognosis in patients with LARC, highlighting its significant potential clinical usefulness.

## AUTHOR CONTRIBUTIONS

**Zhiyuan Zhang:** Conceptualization (lead); data curation (lead); formal analysis (lead); investigation (lead); methodology (lead); project administration (lead); resources (lead); software (equal); validation (equal); visualization (equal); writing – original draft (lead); writing – review and editing (lead). **Jiazhou Wang:** Data curation (lead); formal analysis (lead); funding acquisition (equal); investigation (equal); methodology (lead); resources (equal); software (lead); validation (lead); writing – review and editing (equal). **Di Dai:** Data curation (equal); investigation (equal); resources (equal); validation (lead). **Fan Xia:** Investigation (equal); resources (equal); supervision (equal); writing – review and editing (equal). **Yiqun Sun:** Data curation (equal); investigation (equal); methodology (equal); resources (equal); validation (equal); visualization (equal); writing – review and editing (equal). **Guichao Li:** Resources (equal). **Juefeng Wan:** Resources (equal). **Lijun Shen:** Funding acquisition (equal); resources (equal). **Hui Zhang:** Resources (equal). **Yan Wang:** Resources (equal). **Jie Zhong:** Resources (equal); writing – original draft (equal). **Jun Bao:** Conceptualization (lead); funding acquisition (lead); project administration (equal); resources (lead); visualization (equal); writing – original draft (equal); writing – review and editing (equal). **Zhen Zhang:** Conceptualization (lead); funding acquisition (lead); project administration (lead); resources (lead); supervision (lead); writing – review and editing (lead).

## FUNDING INFORMATION

This work was supported in part by research grants from Shanghai Anticancer Association (HYXH2021096, Zhen Zhang). National Natural Science Foundation of China (82003229, Recipient: Zhen Zhang). The Science and Technology Commission of Shanghai Municipality (STCSM) (21Y21900200, Zhen Zhang). National Natural Science Foundation of China (82272732, Zhen Zhang); Beijing Xisike Clinical Oncology Research Foundation (Y‐Young2022‐0278, Zhiyuan Zhang); Shanghai Municipal Health Commission (20214Y0146, Lijun Shen).

## CONFLICT OF INTEREST STATEMENT

The authors declare no potential conflicts of interest.

## ETHICS STATEMENT

The Research was approved by the Institutional Review Boards of Fudan University Shanghai Cancer Center (2111246‐26) and JiangSu Province Cancer Center (2020科_059), and was conducted under the ethical standards of the 1964 Helsinki Declaration and its later amendments. The trial was approved by the local IRB and participants signed informed consent.

## CODE AVAILABILITY

This radiomic processing software is developed using self‐made code in the R programming language. The relevant code can be referenced from the cited articles in the Methods section of the paper. The code underlying this article may be shared upon reasonable request to the corresponding author, following approval from the involved Research Institutions.

## CONSENT FOR PUBLICATION

Written informed consent was obtained from the patient for publication.

## CONSENT TO PARTICIPATE

Written informed consent was obtained from all patients in this study.

## Supporting information


Data S1:

## Data Availability

The data underlying this article may be shared upon reasonable request to the corresponding author, following approval from the involved Research Institutions.
