# Supplementary material for: Radiomic score for lung nodules as a prognostic biomarker in locally advanced rectal cancer patients: A bi‐institutional study
Source: Cancer Med. 2024 Jun 24;13(12):e7240. doi: 10.1002/cam4.7240 (PMC11196379; doi:10.1002/cam4.7240)
Supplement: Supplementary file 1 — Data S1: [file CAM4-13-e7240-s001.docx]

**Content for supplementary materials**

**Supplementary tables:**

**Supplementary Table S1**

Information for cohorts enrolled and research purposes.

**Supplementary Table S2**

Demographic and clinical characteristics of patients enrolled in development data sets.

**Supplementary Table S3**

Demographic and clinical characteristics of CRC development data set patients in the training set and validation set.

**Supplementary Table S4**

Features selection for radiomic model establishment.

**Supplementary Table S5**

Rad-score distribution characteristics.

**Supplementary Table S6**

Cox regression for clinical prognostic values in FUSCC.

**Supplementary Table S7**

Clinical prognostic values selection in FUSCC cohort by Cox regression.

**Supplementary Table S8**

COX and risk stratification for prognosis.

**Supplementary Figures:**

**Figure S1** Flowchart for radiomic study.

**Figure S2** Illustration of pulmonary nodule ROI delineation.

**Figure S3.** Lasso regression plots.

**Figure S4** Rad-score for patients by different models.

**Figure S5** Calibration curves for the prognostic model.

**Supplementary Table S1** Information for cohorts enrolled and research purposes

|  | CRC patients in development data set  (n=235) | | LARC patients in  FUSCC cohort  (n=169) | | LARC patients in JSCC  cohort  (n=41) | |
| --- | --- | --- | --- | --- | --- | --- |
|  | Metastasis | Benign | Metastasis | Benign | Metastasis | Benign |
| Number of  patients | 121 | 114 | 96 | 73 | 26 | 15 |
| Number of  nodules | 135 | 134 | 116 | 122 | 32 | 37 |
| Pathological  confirmation | 100% | 14.9% | 38.4% | 2.1% | 6.7% | Follow-up  information |
| Lung  metastasis  prediction | Model building for  Rad-score | | Validation for lung  metastasis prediction | | Validation for lung  metastasis prediction | |
| Prognostic  prediction of  Rad-score | - | | Validation for prognostic prediction  of Rad-score | | Validation for prognostic  prediction of Rad-score | |
| Prognostic  prediction of  Cli-Rad-  score | - | | Prognostic model building for Rad- score and clinical  information  comprehensive score | | Validation for prognostic model building for Cli-  Rad-score | |

Characteristics for CRC development data set patients

27

**Supplementary Table S2**

Demographic and clinical characteristics of patients enrolled in development data set

| Characteristic | Patients enrolled in development data set | | | *r* |
| --- | --- | --- | --- | --- |
|  |  | Nodules malignance | |  |
|  | All  (n=235) | Benign  (n = 121) | Metastasis  (n = 114) |  |
| Gender, n (%) |  |  |  | 0.608 |
| Female | 104 (44.3) | 56 (46.3) | 48 (42.1) |  |
| Male | 131 (55.7) | 65 (53.7) | 66 (57.9) |  |
| Age, Median (IQR) | 58.00  [50.00,  63.50] | 60.00  [51.00, 66.00] | 57.00  [50.00, 62.00] | 0.015 |
| Location, n (%) |  |  |  | 0.042 |
| Rectal | 157 (66.8) | 73 (60.3) | 84 (73.7) |  |
| Colon | 78 (33.2) | 48 (39.7) | 30 (26.3) |  |
| Neoadjuvant, n (%) |  |  |  | 0.873 |
| No | 171 (72.8) | 87 (71.9) | 84 (73.7) |  |
| Yes | 64 (27.2) | 34 (28.1) | 30 (26.3) |  |
| Surgery, n (%) |  |  |  | 0.143 |
| No | 1 (0.4) | 0 (0.0) | 1 (0.9) |  |
| Dixon | 125 (53.2) | 59 (48.8) | 66 (57.9) |  |

| Miles | 44 (18.7) | 22 (18.2) | 22 (19.3) |  |
| --- | --- | --- | --- | --- |
| Colon cancer  Resection | 62 (26.4) | 37 (30.6) | 25 (21.9) |  |
| Transanal Resection | 3 (1.3) | 3 (2.5) | 0 (0.0) |  |
| Nodule, n (%) |  |  |  | 0.555 |
| Multi | 88 (37.4) | 48 (39.7) | 40 (35.1) |  |
| Single | 147 (62.6) | 73 (60.3) | 74 (64.9) |  |
| Lateral, n (%) |  |  |  | 0.172 |
| Bilateral | 75 (31.9) | 44 (36.4) | 31 (27.2) |  |
| Unilateral | 160 (68.1) | 77 (63.6) | 83 (72.8) |  |
| CEA, n (%) |  |  |  | 0.015 |
| Normal | 191 (81.3) | 104 (86.0) | 87 (76.3) |  |
| Unnormal | 38 (16.2) | 17 (14.0) | 21 (18.4) |  |
| Unknown | 6 (2.6) | 0 (0.0) | 6 (5.3) |  |
| CA199, n (%) |  |  |  | 0.016 |
| Normal | 198 (84.3) | 104 (86.0) | 94 (82.5) |  |
| Unnormal | 30 (12.8) | 17 (14.0) | 13 (11.4) |  |
| Unknown | 7 (3.0) | 0 (0.0) | 7 (6.1) |  |
| ypT, n (%) |  |  |  | <0.001 |
| ypT0 | 14 (6.0) | 12 (9.9) | 2 (1.8) |  |
| ypT1 | 8 (3.4) | 5 (4.1) | 3 (2.6) |  |
| ypT2 | 48 (20.4) | 28 (23.1) | 20 (17.5) |  |

| ypT3 | 133 (56.6) | 72 (59.5) | 61 (53.5) |  |
| --- | --- | --- | --- | --- |
| ypT4 | 22 (9.4) | 3 (2.5) | 19 (16.7) |  |
| Unknown | 10 (4.3) | 1 (0.8) | 9 (7.9) |  |
| ypN, n (%) |  |  |  | <0.001 |
| ypN0 | 119 (50.6) | 77 (63.6) | 42 (36.8) |  |
| ypN1 | 68 (28.9) | 29 (24.0) | 39 (34.2) |  |
| ypN2 | 36 (15.3) | 13 (10.7) | 23 (20.2) |  |
| Unknown | 12 (5.1) | 2 (1.7) | 10 (8.8) |  |
| yp-stage, n (%) |  |  |  | <0.001 |
| yp0 | 12 (5.1) | 11 (9.1) | 1 (0.9) |  |
| ypI | 32 (13.7) | 25 (20.7) | 7 (6.2) |  |
| ypII | 75 (32.1) | 41 (33.9) | 34 (30.1) |  |
| ypIII | 104 (44.4) | 42 (34.7) | 62 (54.9) |  |
| Unknown | 11 (4.7) | 2 (1.7) | 9 (8.0) |  |
| Pathology, n (%) |  |  |  | 0.084 |
| Adenocarcinoma | 207 (88.1) | 102 (84.3) | 105 (92.1) |  |
| Mucinous  adenocarcinoma | 27 (11.5) | 18 (14.9) | 9 (7.9) |  |
| Signet ring cell  carcinoma | 1 (0.4) | 1 (0.8) | 0 (0.0) |  |
| Differentiation, n (%) |  |  |  | 0.623 |
| Poorly | 29 (12.3) | 15 (12.4) | 14 (12.3) |  |

| Moderately | 147 (62.6) | 79 (65.3) | 68 (59.6) |  |
| --- | --- | --- | --- | --- |
| Highly | 4 (1.7) | 1 (0.8) | 3 (2.6) |  |
| Unknown | 55 (23.4) | 26 (21.5) | 29 (25.4) |  |
| Vascular invasion, n  (%) |  |  |  | <0.001 |
| Negative | 152 (64.7) | 92 (76.0) | 60 (52.6) |  |
| Positive | 39 (16.6) | 21 (17.4) | 18 (15.8) |  |
| Unknown | 44 (18.7) | 8 (6.6) | 36 (31.6) |  |
| PNI, n (%) |  |  |  | <0.001 |
| Negative | 150 (63.8) | 97 (80.2) | 53 (46.5) |  |
| Positive | 41 (17.4) | 16 (13.2) | 25 (21.9) |  |
| Unknown | 44 (18.7) | 8 (6.6) | 36 (31.6) |  |
| CRM, n (%) |  |  |  | <0.001 |
| Negative | 171 (72.8) | 113 (93.4) | 58 (50.9) |  |
| Positive | 3 (1.3) | 0 (0.0) | 3 (2.6) |  |
| Unknown | 61 (26.0) | 8 (6.6) | 53 (46.5) |  |
| Pathologic  confirmation, n (%) |  |  |  | <0.001 |
| No | 103 (43.8) | 103 (85.1) | 0 (0.0) |  |
| Yes | 132 (56.2) | 18 (14.9) | 114 (100.0) |  |

28

29 Characteristics of CRC development data set patients for radiomic training and

30 validation

**Supplementary Table S3**

Demographic and clinical characteristics of CRC development data set patients in the

training set and validation set

| Characteristic | Patients enrolled for model building | | | *r* |
| --- | --- | --- | --- | --- |
|  |  | Nodules distribution | |  |
|  | All(n=246) | Training  (n=173) | Validation  (n=73) |  |
| Lung metastasis, n (%) |  |  |  | 0.577 |
| Benign | 123 (50.0) | 89 (51.4) | 34 (46.6) |  |
| Metastasis | 123 (50.0) | 84 (48.6) | 39 (53.4) |  |
| Gender, n (%) |  |  |  | 0.451 |
| Female | 108 (44.1) | 79 (45.9) | 29 (39.7) |  |
| Male | 137 (55.9) | 93 (54.1) | 44 (60.3) |  |
| Age (median [IQR]) | 58.00  [50.00, 63.00] | 58.00  [50.00, 64.00] | 58.00  [50.75, 63.00] | 0.764 |
| Location, n (%) |  |  |  | 0.969 |
| Rectum | 163 (66.3) | 114 (65.9) | 49 (67.1) |  |
| Colon | 83 (33.7) | 59 (34.1) | 24 (32.9) |  |
| Neoadjuvant, n (%) |  |  |  | 0.832 |
| No | 178 (72.4) | 124 (71.7) | 54 (74.0) |  |

| Yes | 68 (27.6) | 49 (28.3) | 19 (26.0) |  |
| --- | --- | --- | --- | --- |
| Surgery, n (%) |  |  |  | 0.830 |
| No | 2 (0.8) | 2 (1.2) | 0 (0.0) |  |
| Dixon | 130 (52.8) | 92 (53.2) | 38 (52.1) |  |
| Miles | 47 (19.1) | 30 (17.3) | 17 (23.3) |  |
| Colon cancer resection | 63 (25.6) | 46 (26.6) | 17 (23.3) |  |
| Transanal resection | 4 (1.6) | 3 (1.7) | 1 (1.4) |  |
| Nodule, n (%) |  |  |  | 0.628 |
| Single | 151 (61.4) | 104 (60.1) | 47 (64.4) |  |
| Multi | 95 (38.6) | 69 (39.9) | 26 (35.6) |  |
| Lateral, n (%) |  |  |  | 1.000 |
| Unilateral | 165 (67.1) | 116 (67.1) | 49 (67.1) |  |
| Bilateral | 81 (32.9) | 57 (32.9) | 24 (32.9) |  |
| CEA, n (%) |  |  |  | 0.919 |
| Normal | 196 (79.7) | 138 (79.8) | 58 (79.5) |  |
| Unnormal | 41 (16.7) | 28 (16.2) | 13 (17.8) |  |
| Unknown | 9 (3.7) | 7 (4.0) | 2 (2.7) |  |
| CA199, n (%) |  |  |  | 0.704 |
| Normal | 204 (82.9) | 143 (82.7) | 61 (83.6) |  |
| Unnormal | 31 (12.6) | 21 (12.1) | 10 (13.7) |  |
| Unknown | 11 (4.5) | 9 (5.2) | 2 (2.7) |  |
| ypT, n (%) |  |  |  | 0.801 |

| ypT0 | 16 (6.5) | 10 (5.8) | 6 (8.2) |  |
| --- | --- | --- | --- | --- |
| ypT1 | 6 (2.4) | 5 (2.9) | 1 (1.4) |  |
| ypT2 | 44 (17.9) | 30 (17.3) | 14 (19.2) |  |
| ypT3 | 131 (53.3) | 96 (55.5) | 35 (47.9) |  |
| ypT4 | 16 (6.5) | 11 (6.4) | 5 (6.8) |  |
| Unknown | 33 (13.4) | 21 (12.1) | 12 (16.4) |  |
| ypN, n (%) |  |  |  | 0.711 |
| ypN0 | 116 (47.2) | 84 (48.6) | 32 (43.8) |  |
| ypN1 | 66 (26.8) | 43 (24.9) | 23 (31.5) |  |
| ypN2 | 35 (14.2) | 26 (15.0) | 9 (12.3) |  |
| Unknown | 29 (11.8) | 20 (11.6) | 9 (12.3) |  |
| yp-stage, n (%) |  |  |  | 0.750 |
| yp0 | 13 (5.3) | 8 (4.6) | 5 (6.8) |  |
| ypI | 29 (11.8) | 21 (12.1) | 8 (11.0) |  |
| ypII | 73 (29.7) | 55 (31.8) | 18 (24.7) |  |
| ypIII | 101 (41.1) | 69 (39.9) | 32 (43.8) |  |
| Unknown | 30 (12.2) | 20 (11.6) | 10 (13.7) |  |
| Pathology, n (%) |  |  |  | 0.492 |
| Adenocarcinoma | 191 (77.6) | 137 (79.2) | 54 (74.0) |  |
| Mucinous  adenocarcinoma | 27 (11.0) | 19 (11.0) | 8 (11.0) |  |
| Unknown | 28 (11.4) | 17 (9.8) | 11 (15.1) |  |

| Differentiation, n (%) |  |  |  | 0.672 |
| --- | --- | --- | --- | --- |
| Poorly | 28 (11.4) | 17 (9.8) | 11 (15.1) |  |
| Moderately | 140 (56.9) | 101 (58.4) | 39 (53.4) |  |
| Highly | 4 (1.6) | 3 (1.7) | 1 (1.4) |  |
| Unknown | 74 (30.1) | 52 (30.1) | 22 (30.1) |  |
| Vascular invasion, n (%) |  |  |  | 0.297 |
| Negative | 152 (61.8) | 108 (62.4) | 44 (60.3) |  |
| Positive | 41 (16.7) | 25 (14.5) | 16 (21.9) |  |
| Unknown | 53 (21.5) | 40 (23.1) | 13 (17.8) |  |
| PNI, n (%) |  |  |  | 0.651 |
| Negative | 151 (61.4) | 104 (60.1) | 47 (64.4) |  |
| Positive | 42 (17.1) | 29 (16.8) | 13 (17.8) |  |
| Unknown | 53 (21.5) | 40 (23.1) | 13 (17.8) |  |
| CRM , n (%) |  |  |  | 0.947 |
| Negative | 168 (68.3) | 119 (68.8) | 49 (67.1) |  |
| Positive | 3 (1.2) | 2 (1.2) | 1 (1.4) |  |
| Unknown | 75 (30.5) | 52 (30.1) | 23 (31.5) |  |
| Pathologic confirmation,  n (%) |  |  |  | 0.453 |
| No | 105 (42.7) | 77 (44.5) | 28 (38.4) |  |
| Yes | 141 (57.3) | 96 (55.5) | 45 (61.6) |  |

31 Some patients have more than one nodule enrolled in training and validation sets, which

32 cause the repeat count of patients in all number. Finally, 235 patients with 269 nodules 33 were enrolled and 189 nodules were assigned for training and 80 nodules were assigned 34 for validation. CEA Carcinoma Embryonic Antigen, NEI Nerve Invasion, CRM 35 Circumferential Resection Margin, pCR pathological Complete Regression, cN clinical 36 nodal. cT clinical tumor, ypT pathological tumor T stage after neoadjuvant therapy, ypN

37 pathological lymph node stage after neoadjuvant therapy.

38

39

**Supplementary Table S4** Features selection for radiomic model establishment

|  | Feature | Coeficient |
| --- | --- | --- |
| 1 | Intercept | -0.1079374831 |
| 2 | scaled_kurtosis. | 0.2483479160 |
| 3 | LL_GLCM_Maximal_Correlation_Coefficient | -0.1062048366 |
| 4 | HL_GLCM_Sum_variance. | -0.2826984104 |
| 5 | LL_GLRMS_LRHGE. | 0.0935546334 |
| 6 | HH_GLRMS_LGRE. | 0.2280403401 |
| 7 | LH_absolute_median. | -0.0007974185 |
| 8 | HL_absolute_median. | -0.0446257448 |

40

**Supplementary Table S5** Rad-score distribution characteristics

|  | Mean(SD)/ median[IQR] | Cut-off |
| --- | --- | --- |
| Rad-score for CRC training set | 0.46[0.38,0.57] | 0.405 |
| Rad-score for CRC validation set | 0.48(0.11) | 0.518 |
| Rad-score in FUSCC LARC cohort | 0.46[0.38,0.57] | 0.518 |
| Rad-score in JSCC LARC cohort | 0.45[0.42,0.54] | 0.509 |

41

**Supplementary Table S6** Cox regression for clinical prognostic values in FUSCC

| Characteristic | number | Hazard radio | confidence  interval | | *r*-value |
| --- | --- | --- | --- | --- | --- |
| Gender |  |  |  |  |  |
| Female | 62 | - |  |  |  |
| Male | 107 | 0.545 | 0.256 | 1.163 | 0.117 |
| Age | 169 | 1.023 | 0.985 | 1.063 | 0.247 |
| cT |  |  |  |  |  |
| cT3 | 134 | - |  |  |  |
| cT2 | 4 | 0.000 | 0.000 | Infinite | 0.997 |
| cT4 | 31 | 1.335 | 0.534 | 3.335 | 0.537 |
| cN |  |  |  |  |  |
| cN1 | 60 | - |  |  |  |
| cN0 | 9 | 0.000 | 0.000 | Infinite | 0.997 |
| cN2 | 100 | 0.912 | 0.423 | 1.968 | 0.815 |
| CRM |  |  |  |  |  |
| Negative | 42 | - |  |  |  |
| Positive | 21 | 7.571 | 1.448 | 39.579 | 0.016 |
| Unknown | 106 | 1.906 | 0.427 | 8.507 | 0.398 |
| cEMVI |  |  |  |  |  |
| Negative | 30 | - |  |  |  |
| Positive | 16 | 1.617 | 0.269 | 9.716 | 0.599 |

| Unknown | 123 | 0.785 | 0.222 | 2.772 | 0.706 |
| --- | --- | --- | --- | --- | --- |
| cstage |  |  |  |  |  |
| cI | 2 | 0.000 | 0.000 | Infinite | 0.997 |
| cII | 7 | 0.000 | 0.000 | Infinite | 0.998 |
| cIII | 160 | - |  |  |  |
| CEA | |  |  |  |  |
| Normal | 96 | - |  |  |  |
| Abnormal | 70 | 1.462 | 0.686 | 3.112 | 0.325 |
| Unknown | 3 | 0.000 | 0.000 | Infinite | 0.998 |
| Distance to anus | |  |  |  |  |
| Lower | 95 | - |  |  |  |
| Middle | 74 | 0.698 | 0.319 | 1.526 | 0.368 |
| Surgery type |  |  |  |  |  |
| Dixon | 91 | - |  |  |  |
| Miles | 72 | 1.349 | 0.632 | 2.881 | 0.439 |
| Hartmann | 6 | 0.000 | 0.000 | Infinite | 0.997 |
| TRG |  |  |  |  |  |
| TRG0 | 28 | - |  |  |  |
| TRG1 | 38 | 2.164 | 0.435 | 10.757 | 0.345 |
| TRG2 | 81 | 3.268 | 0.743 | 14.367 | 0.117 |
| TRG3 | 17 | 2.996 | 0.499 | 17.990 | 0.230 |
| Unknown | 5 | 3.443 | 0.312 | 38.039 | 0.313 |

| pCR |  |  |  |  |  |
| --- | --- | --- | --- | --- | --- |
| Non-pCR | 142 | - |  |  |  |
| pCR | 24 | 0.220 | 0.030 | 1.630 | 0.138 |
| Unknown | 3 | 1.973 | 0.266 | 14.643 | 0.506 |
| Maximum tumor  diameter | 169 | 1.087 | 0.748 | 1.578 | 0.663 |
| Pathology |  |  |  |  |  |
| Adenocarcinoma | 159 | - |  |  |  |
| Mucinous  adenocarcinoma | 4 | 1.080 | 0.145 | 8.051 | 0.940 |
| Unknown | 6 | 0.000 | 0.000 | Infinite | 0.997 |
| Differentiation | |  |  |  |  |
| Pooly | 17 | - |  |  |  |
| Moderately | 82 | 0.429 | 0.152 | 1.215 | 0.111 |
| Highly | 4 | 0.000 | 0.000 | Infinite | 0.997 |
| Unknown | 66 | 0.303 | 0.100 | 0.918 | 0.035 |
| CRM |  |  |  |  |  |
| Negative | 165 | - |  |  |  |
| Positive | 4 | 3.897 | 0.893 | 17.011 | 0.070 |
| Vascular invasion |  |  |  |  |  |
| Negative | 155 | - |  |  |  |
| Positive | 14 | 1.902 | 0.643 | 5.620 | 0.245 |

| PNI |  |  |  |  |  |
| --- | --- | --- | --- | --- | --- |
| Negative | 144 | - |  |  |  |
| Positive | 25 | 2.260 | 0.940 | 5.434 | 0.069 |
| Cut margin |  |  |  |  |  |
| Negative | 168 | - |  |  |  |
| Positive | 1 | 0.000 | 0.000 | Infinite | 0.997 |
| ypT |  |  |  |  |  |
| ypT0 | 29 | - |  |  |  |
| ypT1 | 6 | 0.000 | 0.000 | Infinite | 0.997 |
| ypT2 | 45 | 2.103 | 0.423 | 10.453 | 0.364 |
| ypT3 | 86 | 3.683 | 0.851 | 15.943 | 0.081 |
| ypT4 | 3 | 5.873 | 0.530 | 65.095 | 0.149 |
| YpN |  |  |  |  |  |
| ypN0 | 103 | - |  |  |  |
| ypN1 | 52 | 3.792 | 1.651 | 8.710 | 0.002 |
| ypN2 | 14 | 2.064 | 0.549 | 7.761 | 0.283 |
| yp-stage |  |  |  |  |  |
| yp0-I | 62 | - |  |  |  |
| ypII | 39 | 3.405 | 0.851 | 13.627 | 0.083 |
| ypIII | 68 | 6.020 | 1.771 | 20.458 | 0.004 |
| Number of lung nodules |  |  |  |  |  |
| Single | 87 | - |  |  |  |

| Multi | 82 | 1.854 | 0.839 | 4.094 | 0.127 |
| --- | --- | --- | --- | --- | --- |

The control group for Cox regression in each clinical value was labeled by ‘- ’symbol. And because of the limited number of outcome events in the subgroup, the HR and interval were calculated as infinite, with an uncertain *P-*value, LARC Locally Advanced Rectal Cancer, FUSCC Fudan University Cancer Center, CEA Carcinoma Embryonic Antigen, PNI peirneural invasion, CRM Circumferential Resection Margin, pCR pathological Complete Regression, cN clinical nodal. cT clinical tumor, ypT pathological tumor T stage after neoadjuvant therapy, ypN pathological lymph

node stage after neoadjuvant therapy.

42

**Supplementary Table S7** Clinical prognostic values selection in FUSCC cohort by

Cox regression

| Characteristic | Hazard radio | confidence interval | | *r*-value |
| --- | --- | --- | --- | --- |
| CRM | 4.178 | 0.797 | 21.897 | 0.091 |
| PNI | 1.064 | 0.388 | 2.919 | 0.905 |
| yp-stage 0-I | - |  |  |  |
| yp-stage II | 2.838 | 0.680 | 11.845 | 0.153 |
| yp-stage III | 5.958 | 1.694 | 20.955 | 0.005 |

CRM, Circumferential Resection Margin, was excluded because of the number of known events is limited. PNI, Perineural Invasion. Every element was selected based on the P-value<0.1, andyp-stage was the final clinical characteristic selected

for further Rad-clinical model establishment.

43

**Supplementary Table S8** COX and risk stratification for prognosis

| Model |  | Hazard  Ratio | 95% confidence  interval | *P-*value |
| --- | --- | --- | --- | --- |
| Prognostic prediction by  ypTNM stage | Stage I | - | - | - |
|  | Stage II | 2.168 | 0.785-5.985 | 0.135 |
|  | Stage III | 3.498 | 1.526-8.017 | 0.003 |
| Prognostic prediction by  Rad-score |  | 1.043 | 1.010- 1.077 | 0.011 |
| Prognostic prediction by  Cli-Rad-score | Stage I | - | - | - |
|  | Stage II | 1.954 | 0.702-5.437 | 0.199 |
|  | Stage III | 3.335 | 1.454-7.647 | 0.004 |
|  | Rad-score | 1.041 | 1.007- 1.077 | 0.017 |
| Risk stratification |  |  |  |  |
| Risk stratification by ypTNM stage in FUSCC  cohort |  | 2.497 | 1.365-4.569 | 0.003 |
| Risk stratification by ypTNM stage in JSCC  cohort |  | 3.462 | 1.211-9.899 | 0.007 |
| Risk stratification by Rad-  score in FUSCC cohort |  | 2.884 | 1.523-5.459 | <0.001 |

| Risk stratification by Rad-  score in JSCC cohort |  | 3.341 | 0.858- 13.000 | 0.010 |
| --- | --- | --- | --- | --- |
| Risk stratification by Cli- Rad-score in FUSCC  cohort |  | 3.544 | 1.596-7.870 | <0.001 |
| Risk stratification by Cli-  Rad-score in JSCC cohort |  | 4.751 | 1.720- 13.120 | 0.003 |

44

| **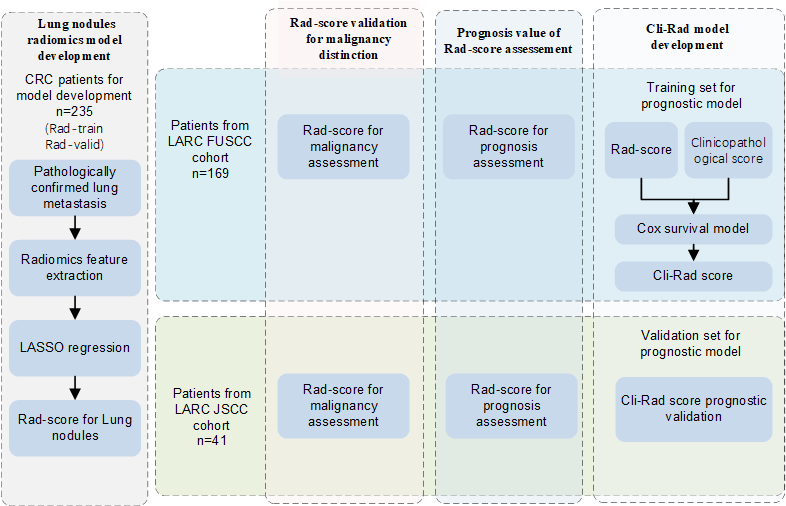** |
| --- |
| **Figure S1** Flowchart for radiomic study.  The lung images from CRC patients in Fudan University Shanghai Cancer Center were divided into two cohorts at a ratio of 7:3 to generate a radiomic model by LASSO regression and validate it. To test the stability of the model in LARC patients, the malignant prediction from the radiomic model was conducted in two separate cohorts from FUSCC (FUSCC cohort) and JiangSu province Cancer Center (JSCC cohort). To explore the prognostic value of the radiomic model in LARC patients, a COX regression model was built based on FUSCC cohort, and then a nomogram based on the clinical and radiomic information was built in FUSCC cohort and validated in JSCC cohort.  CRC colon and rectal cancer, FUSCC Fudan University Shanghai Cancer Center, JSCC JiangSu province Cancer Center, LASSO Least Absolute Shrinkage and Selection Operator, LARC Locally Advanced Rectal Cancer. |

**Supplementary figures**

| **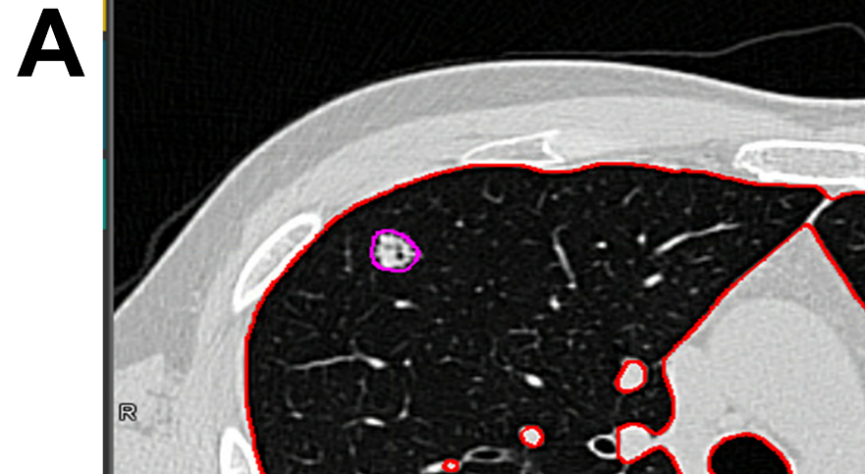** |
| --- |
| **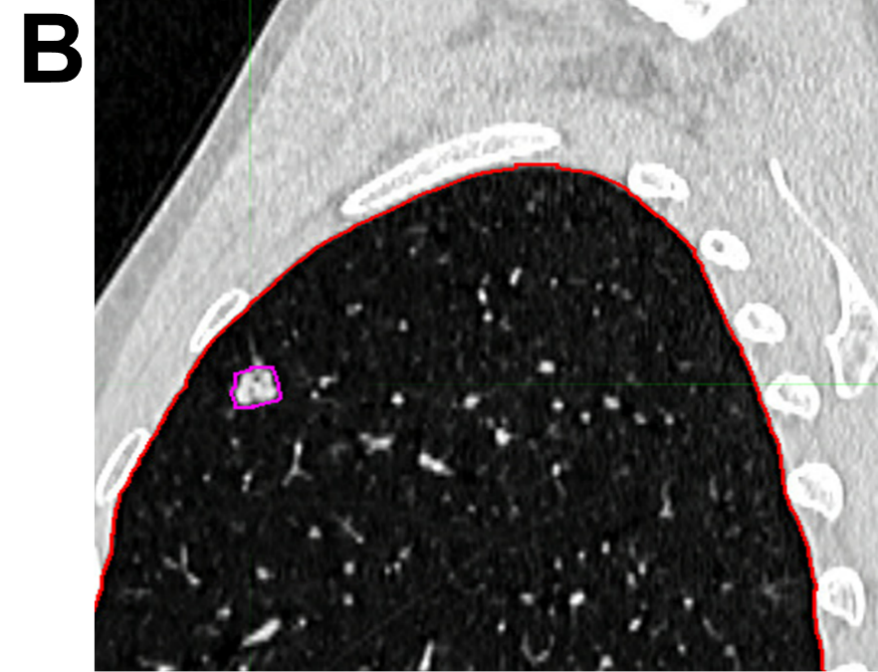** |
| **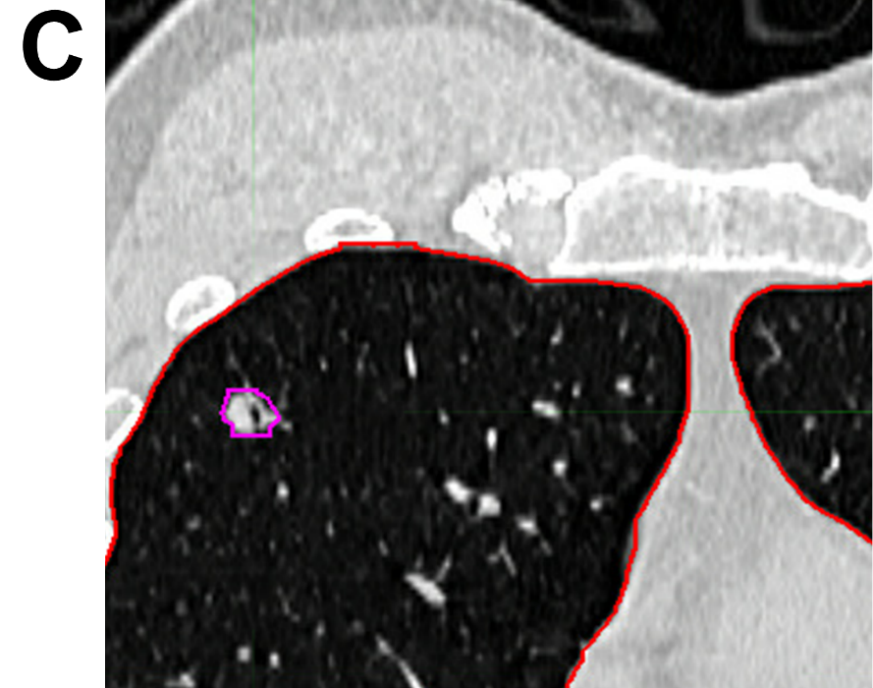** |
| **Figure S2** Illustration of pulmonary nodule ROI delineation.  (A) represents the morphology of pulmonary nodules in the transverse plane. (B)represents the morphology of pulmonary nodules in the sagittal plane. (C) represents the morphology of pulmonary nodules in the coronal plane. |

| 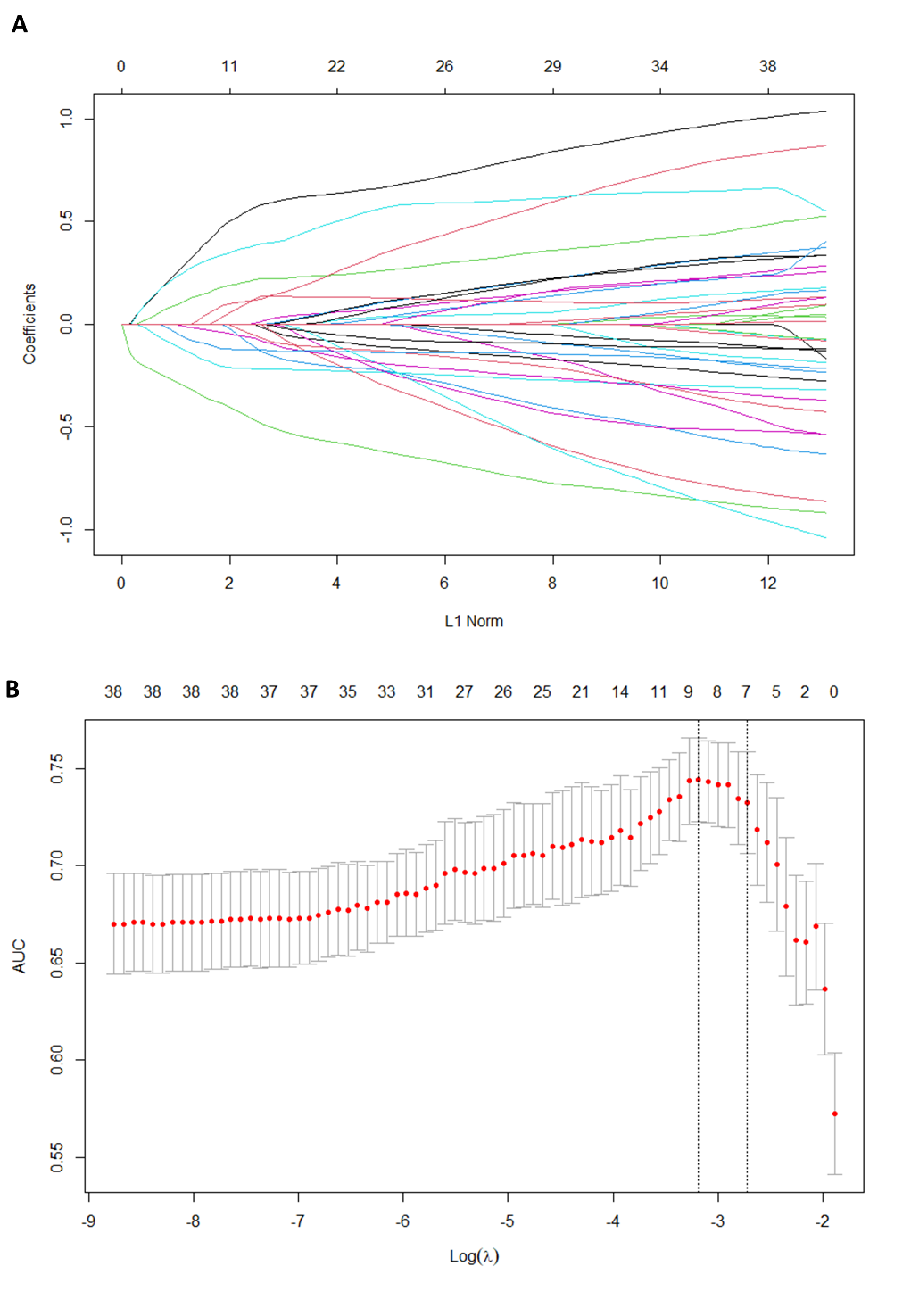 |
| --- |
| **Figure S3** Lasso regression plots.  Lasso regression plots were listed above. (A) Text features were selected by the LASSO regression model. The performance of the radiomics signature was assessed by the ROC curve and C-index. Tuning parameter (λ) selection used ten-fold cross-validation via the minimum criteria. The optimal value was calculated by the minimum criteria and the 1-standard error of the minimum criteria (the 1-SE criteria). λ of 0.0661 with SE of 0.2617 was chosen. (B) A LASSO coefficient profile plot was produced against the log(λ) sequence. In addition, seven radiomics feature was selected. |

| 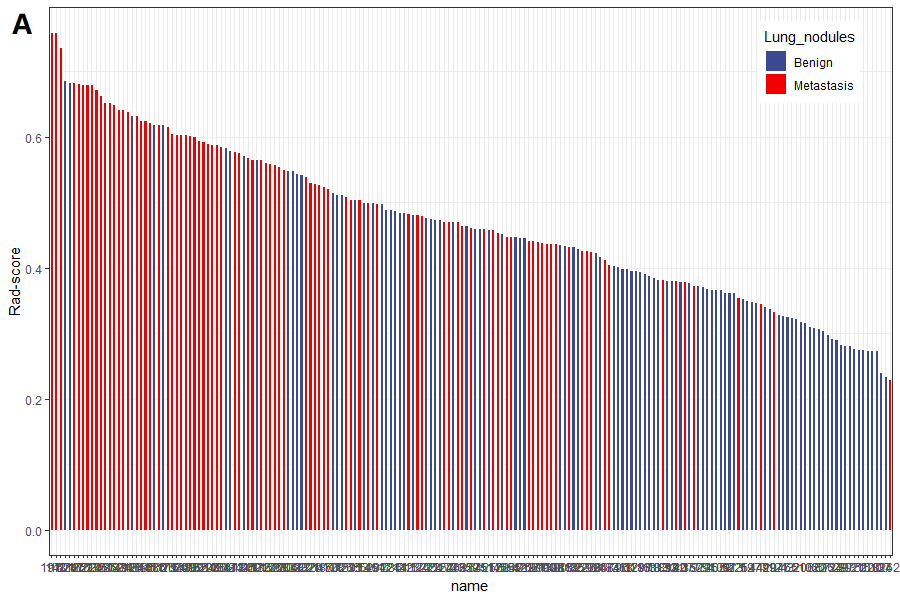  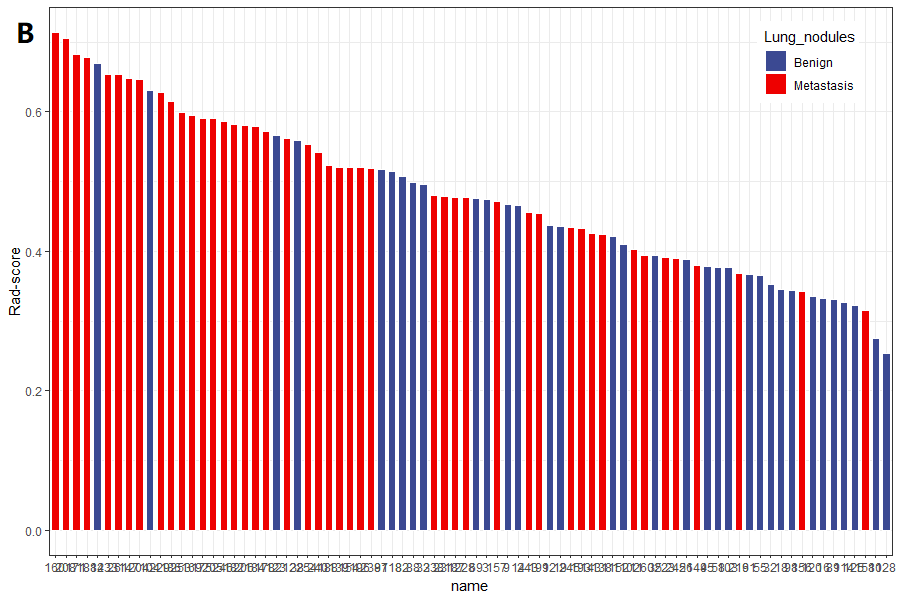  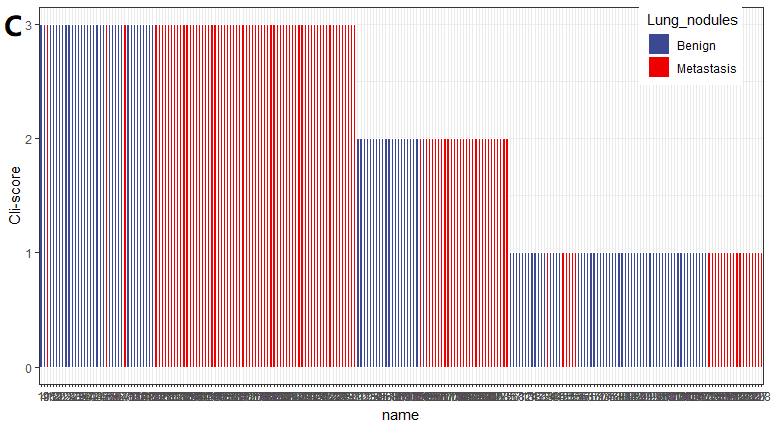  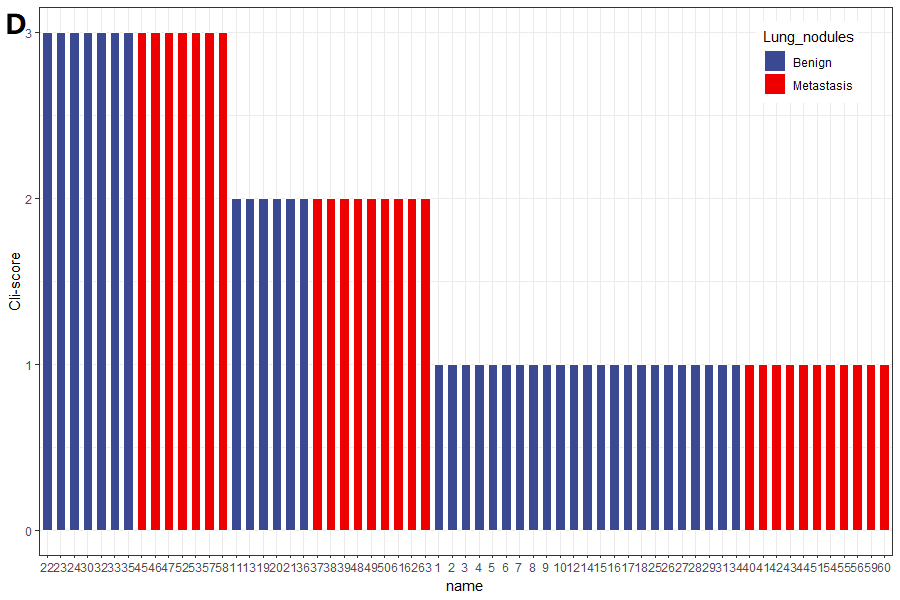  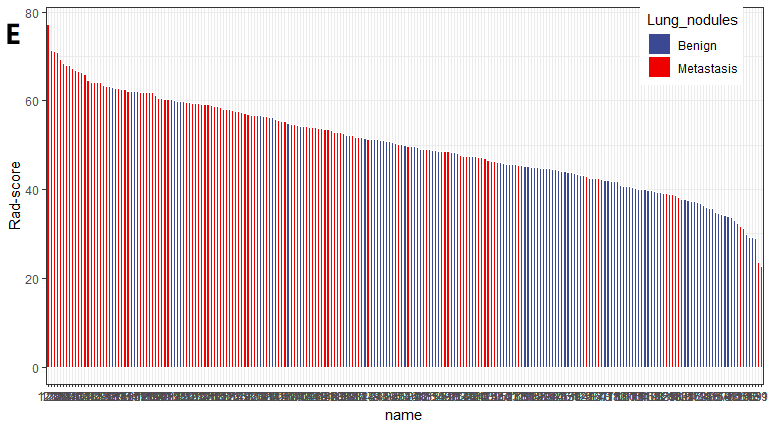  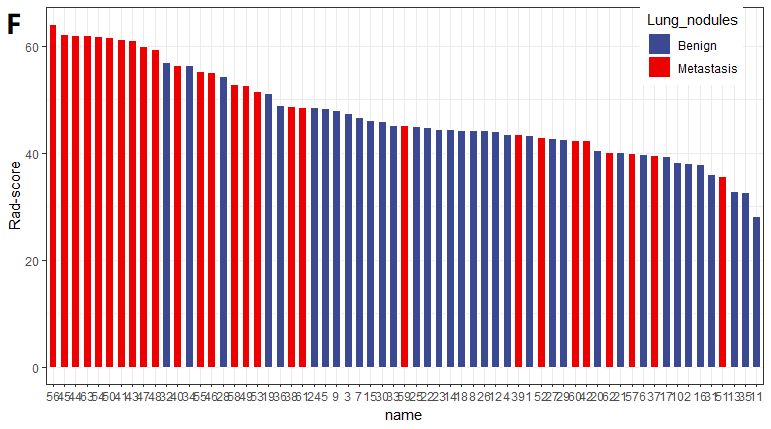  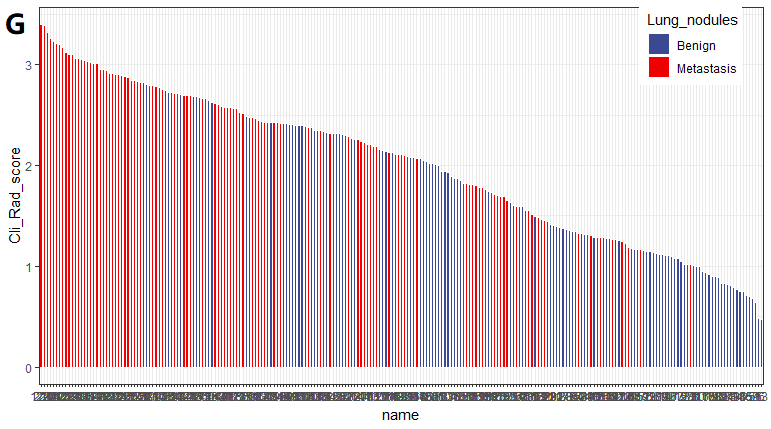  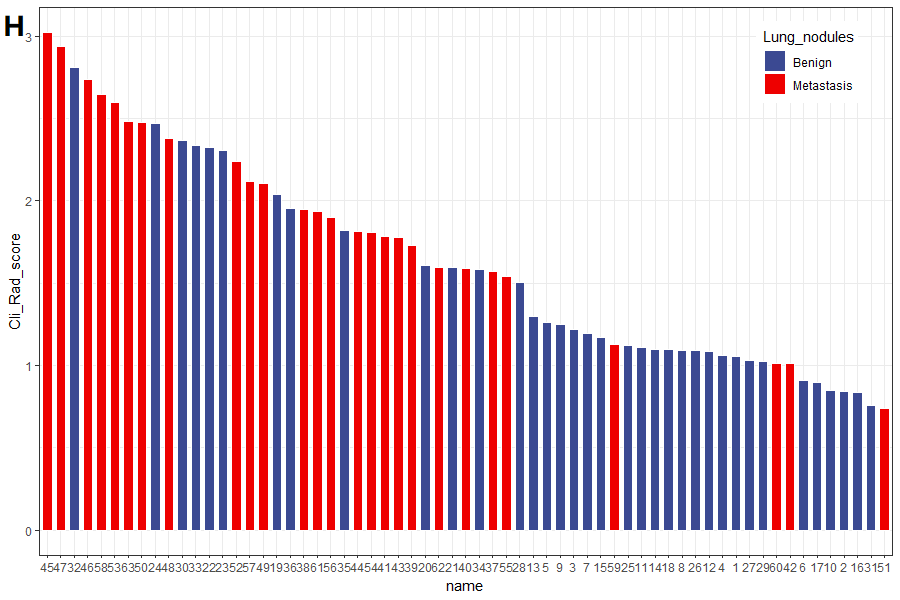 |
| --- |
| **Figure S4** Rad-score for patients by different models.  (A) represented CRC patients for radiomic model establishment from the training set. (B) represented CRC patients from the validation set. (C) represented the score from the ypTNM stage for LARC patients from LARC internal cohort. (D) represented the score from the ypTNM stage for LARC patients from LARC external cohort. (E) represented the Rad-score for LARC patients with lung nodules in LARC internal cohort. (F) represented the Rad-score for LARC patients with lung nodules in LARC external cohort. (G) represented the Clinical-Rad-score for LARC patients with lung nodules in LARC internal cohort, and (H) represented the Clinical-Rad-score for LARC patients with lung nodules in LARC external cohort. |

| 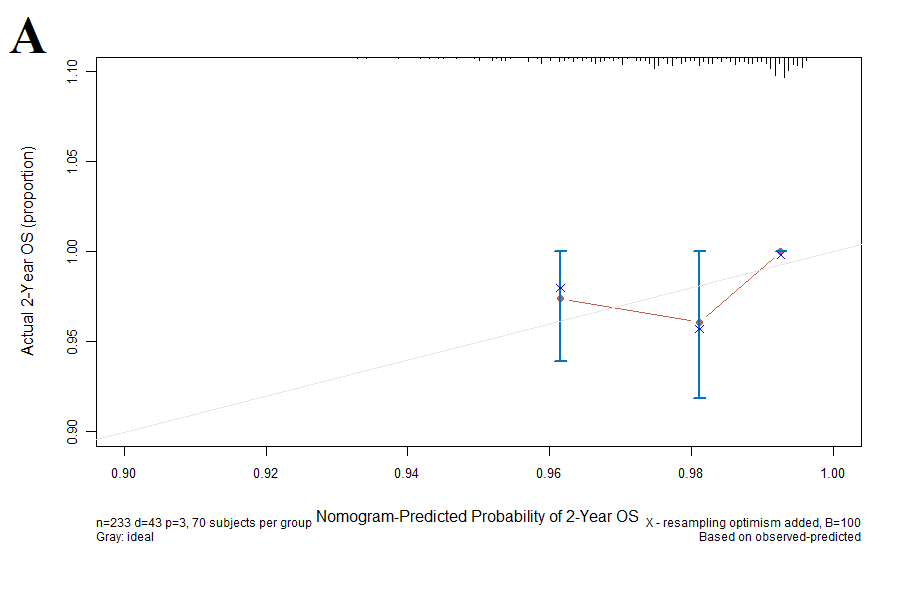  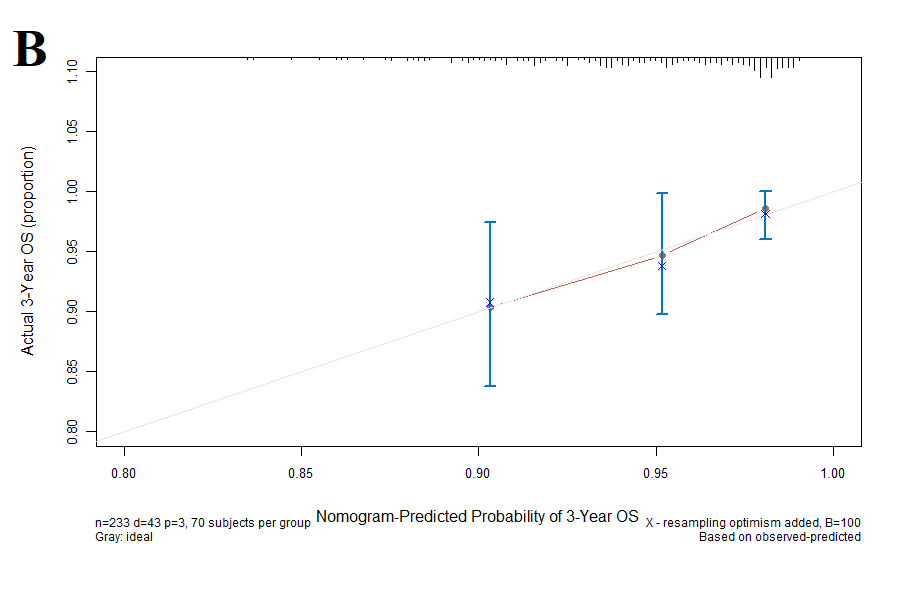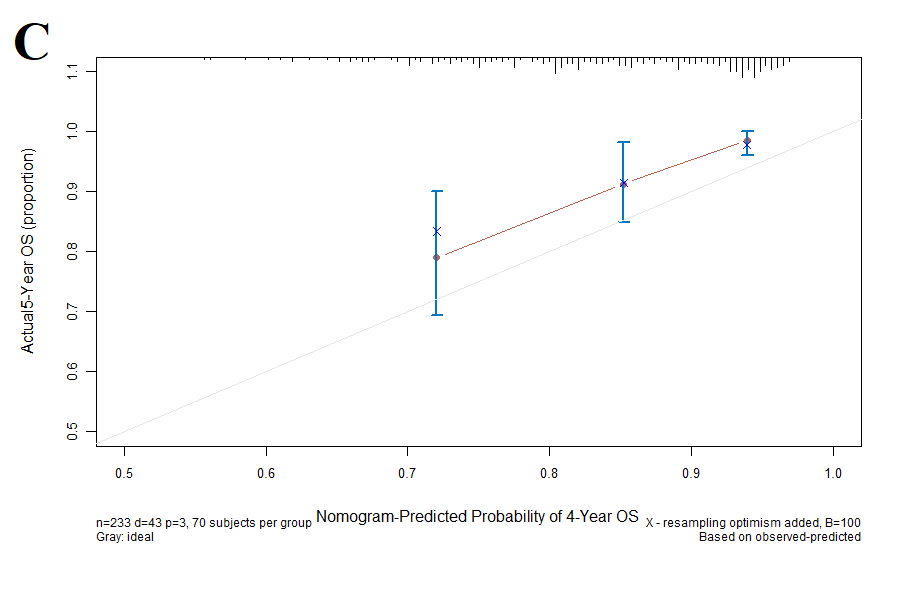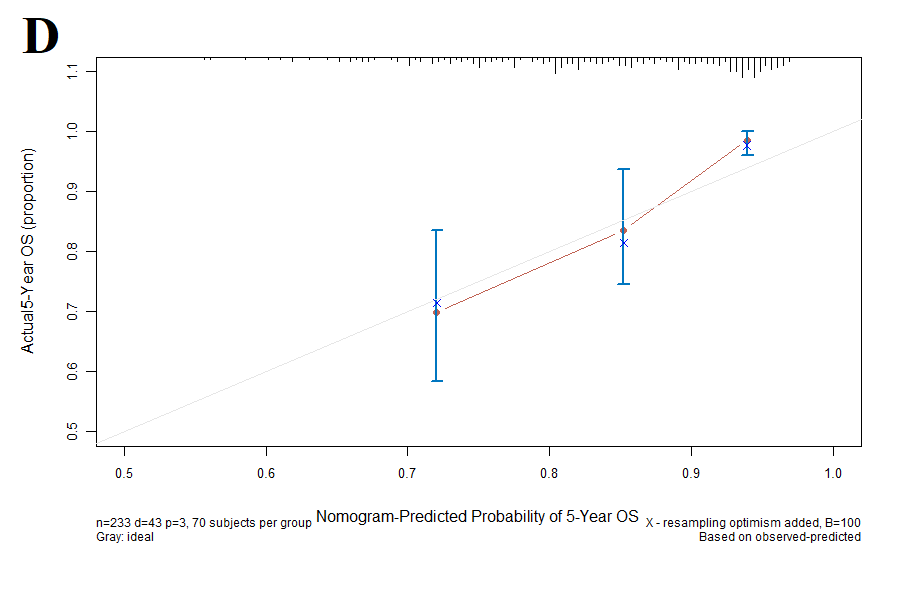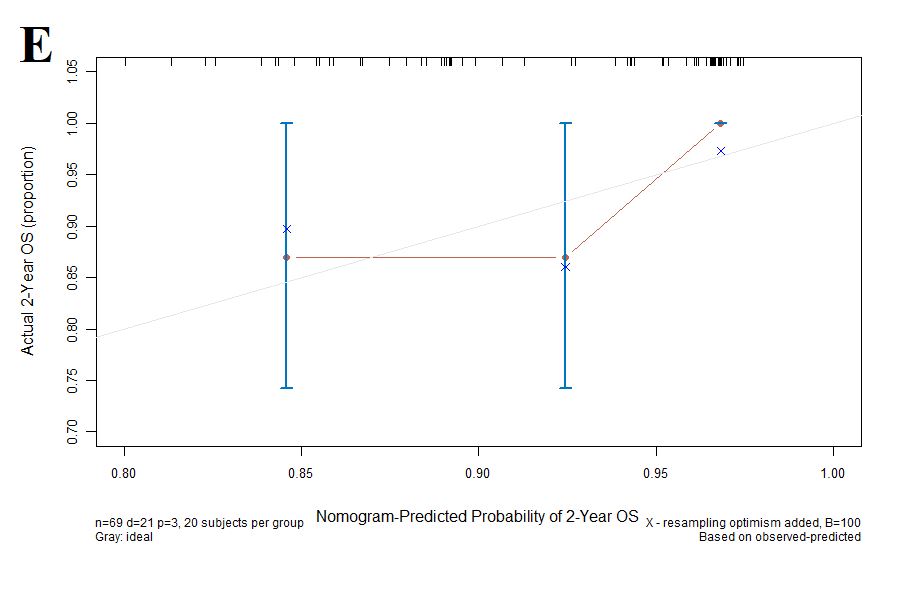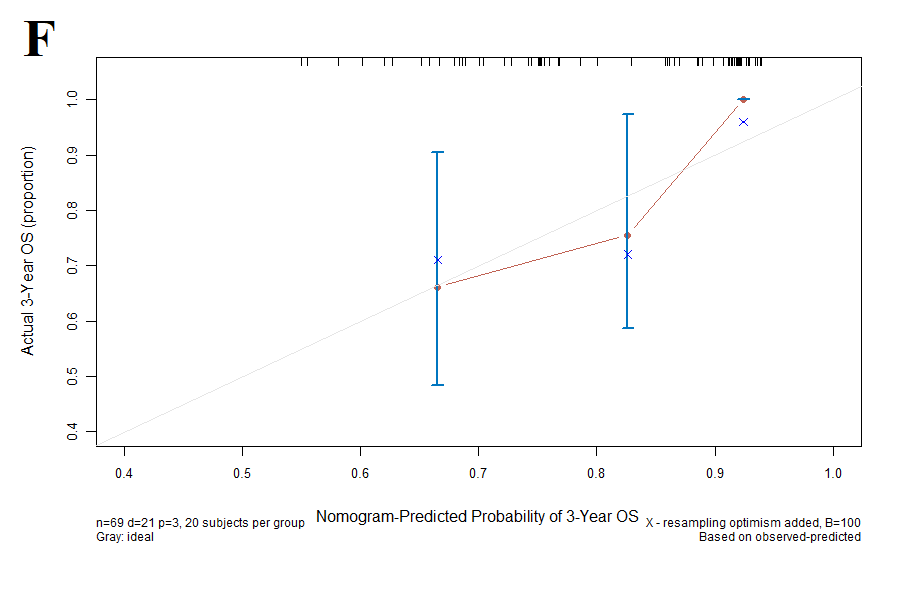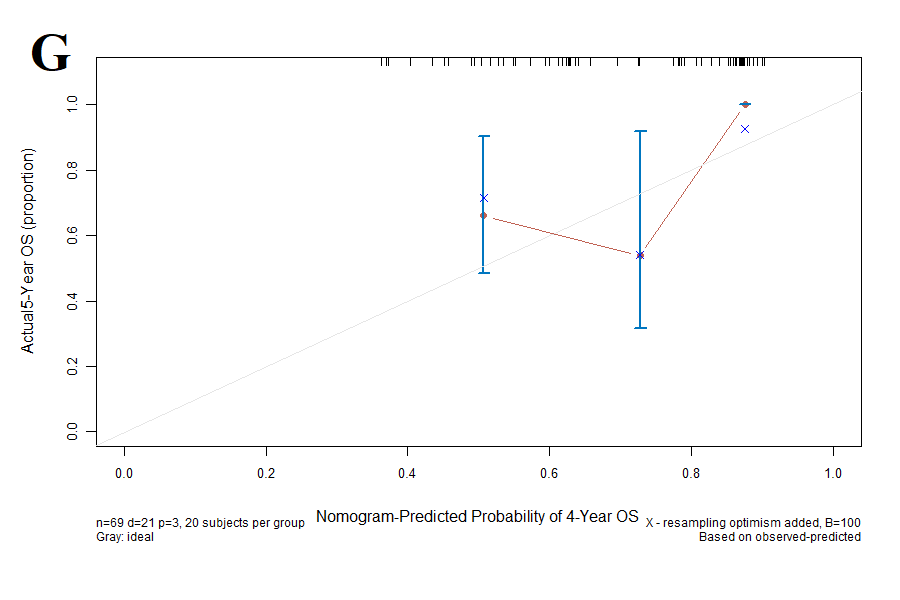 |
| --- |
| **Figure S5** Calibration curves for the prognostic model.  (A) represented the time point of 24 months from LARC internal cohort, (B) represented the time point of 36 months from LARC internal cohort, (C) represented the time point of 48 months from LARC internal cohort, (D) represented the time point of 60 months from LARC internal cohort. And (E) represented the time point of 24 months from LARC external cohort, (F) represented the time point of 36 months from LARC external cohort, (G) represented the time point of 48 months from LARC external cohort. |

49
